# Characterization of the nucleotide-binding domain NsrF from the BceAB-type ABC-transporter NsrFP from the human pathogen *Streptococcus agalactiae*

**DOI:** 10.1038/s41598-020-72237-7

**Published:** 2020-09-16

**Authors:** Fabia Furtmann, Nicola Porta, Dai Tri Hoang, Jens Reiners, Julia Schumacher, Julia Gottstein, Holger Gohlke, Sander H. J. Smits

**Affiliations:** 1 Institute of Biochemistry, Heinrich-Heine-Universität Düsseldorf, Universitätsstraße 1, 40225 Düsseldorf, Germany; 2 Institute for Pharmaceutical and Medicinal Chemistry, Heinrich-Heine-Universität Düsseldorf, Universitätsstraße 1, 40225 Düsseldorf, Germany; 3 Center for Structural Studies, Heinrich-Heine-Universität Düsseldorf, Universitätsstraße 1, 40225 Düsseldorf, Germany; 4 John von Neumann Institute for Computing (NIC), Jülich Supercomputing Centre (JSC), Institute of Biological Information Processing (IBI-7: Structural Biochemistry),, Forschungszentrum Jülich GmbH, Wilhelm-Johnen-Straße, 52425 Jülich, Germany

**Keywords:** Protein structure predictions, Hydrolases, Molecular modelling, SAXS, Nucleotide-binding proteins, Biochemistry

## Abstract

Treatment of bacterial infections is a great challenge of our era due to the various resistance mechanisms against antibiotics. Antimicrobial peptides are considered to be potential novel compound as antibiotic treatment. However, some bacteria, especially many human pathogens, are inherently resistant to these compounds, due to the expression of BceAB-type ABC transporters. This rather new transporter family is not very well studied. Here, we report the first full characterization of the nucleotide binding domain of a BceAB type transporter from *Streptococcus agalactiae*, namely *Sa*NsrF of the transporter *Sa*NsrFP*,* which confers resistance against nisin and gallidermin. We determined the NTP hydrolysis kinetics and used molecular modeling and simulations in combination with small angle X-ray scattering to obtain structural models of the *Sa*NsrF monomer and dimer. The fact that the *Sa*NsrF_H202A_ variant displayed no ATPase activity was rationalized in terms of changes of the structural dynamics of the dimeric interface. Kinetic data show a clear preference for ATP as a substrate, and the prediction of binding modes allowed us to explain this selectivity over other NTPs.

## Introduction

Therapeutic compounds against bacterial infections are currently one of the biggest needs worldwide. Among antibiotics, antimicrobial peptides (AMP) offer promising potential for the treatment of bacterial infections, alone or in combination with already known molecules^1,2^. An alarming number of pathogenic multidrug resistant strains have evolved under the selective pressure caused by decades of incorrect antibiotic usage. Among them, methicillin-resistant *Staphylococcus aureus* (MRSA) or vancomycin-resistant *Enterococcus* (VRE) pose a high risk to therapeutic regimens^3^. To include new classes of antibiotics in therapy, studies were performed with lantibiotics, a class of AMPs. These ribosomally-synthesized peptides exhibit high potency against several human pathogenic bacterial strains^2–4^ and show high stability to chemical and enzymatic degradation due to multiple intramolecular thioether rings and unsaturated amino acids^4–8^.

Most known lantibiotics act similar in that they inhibit cell wall synthesis^9^. A common target for AMPs is the peptidoglycan layer, which exists in Gram-positive as well as Gram-negative bacteria. It is built up by altering amino sugars such as *N*-acetylglucosamine (GlcNAc) and *N*-acetylmuramic acid (MurNAc) and stabilized by a cross-linkage of those polymer chains. The inhibition of the cell wall synthesis results in reduced cell growth and subsequent cell death. The well-known lantibiotic nisin contains five lanthionine rings and primarily targets the cell wall precursor Lipid II. The initial binding of the first two N-terminal lanthionine rings (A and B) of the lantibiotic to Lipid II is followed by a reorientation of the C-terminus into the membrane, resulting in pore formation and subsequently cell lysis^10,11^. Even though lantibiotics are effective in the nanomolar range, their application is hampered by resistance-conferring mechanisms found in human pathogenic bacteria^7,12,13^. The resistance is mediated by a newly discovered class of ATP binding cassette transporters, called Bacitracin efflux ABC transporters (BceAB), named after their first discovery in the bacitracin resistant strain of *Bacillus subtilis*^14,15^. In *Streptococcus agalactiae* such a BceAB-type ABC transporter is also present, as part of an operon that confers resistance against the lantibiotic nisin^16^. This operon consists of the membrane-associated protease *Sa*Nsr^17^, the ABC transporter *Sa*NsrFP^8^, and the two-component system comprising the response regulator *Sa*NsrR and the histidine kinase *Sa*NsrK^18^. So far, structural information is known only for *Sa*Nsr^17^ and *Sa*NsrR^18^.

Like all ABC transporters, BceAB-type transporters are composed of a nucleotide-binding domain (NBD) and a transmembrane domain (TMD). The NBD hydrolyses ATP, which drives conformational changes in the TMD, leading to substrate translocation. The TMD of BceAB-type ABC transporters are characterized by ten predicted transmembrane helices and a large extracellular domain (ECD_L_) of ~ 220 amino acids that is the hallmark of this transporter family^4,8,16^.

Sequences of the TMD domains from various BceAB-type ABC transporters are not very similar, which explains the large variety of substances they are able to translocate^16^. In contrast, NBDs share sequence and distinct motifs which are highly conserved throughout the ABC transporter superfamily^19–22^. NBDs are mainly L-shaped and comprise a helical signaling domain and a catalytic domain built of α-helices and β-strands^23–25^. The catalytic domain contains the Walker A motif that forms the nucleotide-binding site. A glutamate residue in the Walker B motif takes part in proper nucleotide binding; the γ-phosphate of the ATP molecule is sensed by a conserved histidine (H-loop) which when mutated results in an inactive variant^22,23,26^. Signaling and catalytic domains are connected by the Q- and the P-loop. Within the signaling domain the C-loop is located, which is the signature motif of an ABC transporter (for an alignment see Fig. S6 and Table S2)^22,27,28^.

Dimerization of two NBD monomers in a head-to-tail conformation, is needed to enable ATP hydrolysis with the nucleotide binding sites located in the dimer interface. Each ATP molecule is sandwiched between the Walker A motif of one monomer and the C-loop of the second one, which results in a closed, stable complex^24,29–31^. An interaction between the NBD and the nucleotide is supposed to occur by π–π-stacking between the aromatic ring system of the nucleotide and an aromatic residue of the protein (F or Y). Hence, no preference towards any nucleotide-triphosphate (NTP) has been assumed^24^, as also observed for example for yeast PDR5^32^. The hydrolysis of ATP is coupled to the presence of a cofactor, almost exclusively Mg^2+^, which is coordinated by the Walker B motif. The divalent cation participates in the hydrolytic attack on the γ-phosphate of the nucleotide^26,28,31^.

Here, we report for the first time biochemical and structural characteristics of the BceA nucleotide binding domain *Sa*NsrF, through NTP hydrolysis assays, molecular modeling and simulations. *Sa*NsrF is part of the BceAB-type ABC transporter NsrFP from *Streptococcus agalactiae*^16^. We show that the NBD *Sa*NsrF_WT_ and its hydrolysis-deficient variant *Sa*NsrF_H202A_ are monomeric in solution. Broad-ranging in vitro ATPase screenings delivered detailed information about the protein’s properties with regard to its structure and physiology. We show that the preferred substrate of *Sa*NsrF is ATP as demonstrated by its kinetic parameters. Moreover, we built a structural model of the ATP/Mg^2+^-bound *Sa*NsrF protein in its monomeric and dimeric form by comparative modeling and molecular dynamics simulations. In all, this constitutes the first biochemical characterization of a BceAB-type NBD.

## Results

### Cloning, expression and purification

**Figure 1:**
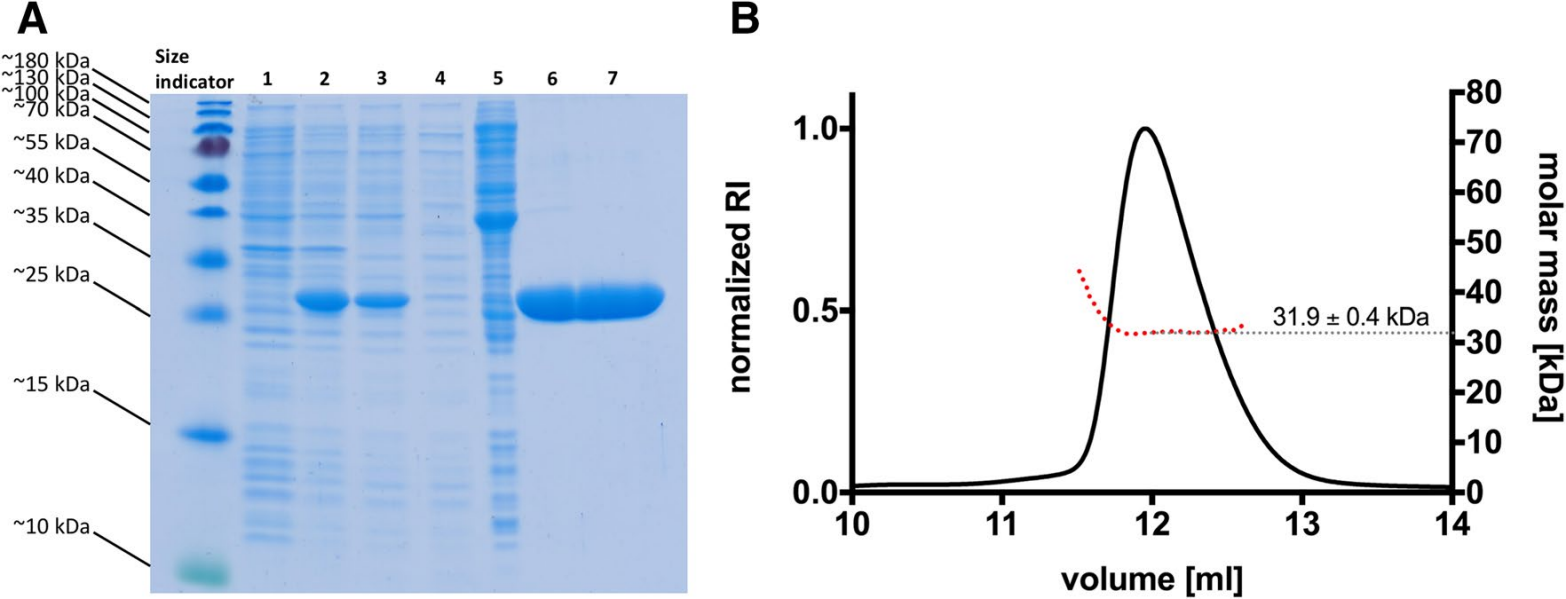
Purification and SEC-MALS of *Sa*NsrF_WT_. (**A**) SDS-PAGE of the *Sa*NsrF_WT_ purification progress. PageRuler Prestained Protein Ladder (size indicator; 10 to 180 kDa), *E. coli* strain before IPTG induction (1), *E. coli* strain after IPTG induction (2), IMAC load (3), IMAC flow-through (4), IMAC wash-fraction (5), IMAC eluate (6), SEC eluate (7). (**B**) Multiangle Light Scattering of *Sa*NsrF_WT_. Freshly purified *Sa*NsrF_WT_ was diluted in MALS-buffer and applied with a concentration of 3 mg mL^−1^ onto a Superdex 75 16/300 increase column. MALS-RI analysis shows that the *Sa*NsrF_WT_ protein elutes with an absolute molecular mass of 31.9 ± 0.4 kDa, consistent with a theoretical monomeric mass in solution.

For substrate transport BceAB-type ABC transporters depend on energy supply generated by ATP hydrolysis, which is mediated by the NBD. Here, we characterized the NBD NsrF of the BceAB-type ABC transporter NsrFP from *Streptococcus agalactiae*. To heterologously express *Sa*NsrF_WT_ and *Sa*NsrF_H202A_, we constructed expression vectors using a codon-optimized version of *Sa*NsrF for the heterologous expression in *E. coli* (Gen Bank accession number: WP_000923537). These constructs expressed a *Sa*NsrF protein with an N-terminal His10-tag attached for purification using Metal Ion Affinity Chromatography. The corresponding *Sa*NsrF constructs were expressed under the control of the plasmid-based T7-promoter via induction with Isopropyl-β-D-thiogalactopyranoside (IPTG). *Sa*NsrF_WT_ was purified to high homogeneity (Fig. 1A), and was examined by Size Exclusion Chromatography coupled to Multiangle Light Scattering (SEC-MALS)^33^, which revealed a molecular mass of 31.9 ± 0.4 kDa for the *Sa*NsrF_WT_ protein (Fig. 1B). This corresponds nicely with the calculated theoretical molecular mass of the recombinant monomer of 30.9 kDa including the His10-tag. Thus, the conducted SEC-MALS analysis revealed that *Sa*NsrF_WT_ exists as a stable monomer in solution, which is in line with previous observations of other NBDs from different ABC transporter families^34–36^.

By sequence alignments, His_202_ was identified to be the essential residue of the H-loop^37–39^. As shown for other NBDs, a point mutation to alanine results in a loss of the ATPase activity of the NBD. We generated this variant of *Sa*NsrF (*Sa*NsrF_H202A_), which indeed displayed no NTP hydrolysis (see below). This variant served as a negative control in all our experiments. The lack of NTP hydrolysis for *Sa*NsrF_H202A_ is in line with in vivo studies that show that this variant abolishes the activity of *Sa*NsrFP^8,40^.

### Activity of SaNsrF_WT_

After successful purification, we functionally characterized *Sa*NsrF_WT_. To do so, we screened the following parameters for their influence on the ATP hydrolysis velocity: (I) pH, (II) salt concentration, (III) nature of the divalent ion and (IV) temperature (see Supporting Information and Fig. S1). As a result, the optimized conditions were found to be 100 mM HEPES at pH 7 with 0 mM NaCl as an assay buffer. The buffer included 10 mM Mg^2+^ and the reaction was finally performed at 30 °C, with an incubation time of 18 min (Fig S1). These optimized conditions were applied in all following experiments.

### Velocity of NTP hydrolysis by SaNsrF_WT_ and SaNsrF_H202A_

Kinetic measurements were performed by quantifying the NTP hydrolysis under increasing concentrations of the respective nucleotide. We determined the NTP hydrolysis behaviour of *Sa*NsrF_WT_ and *Sa*NsrF_H202A_ using increasing amounts of ATP, GTP, CTP or UTP.

**Figure 2:**
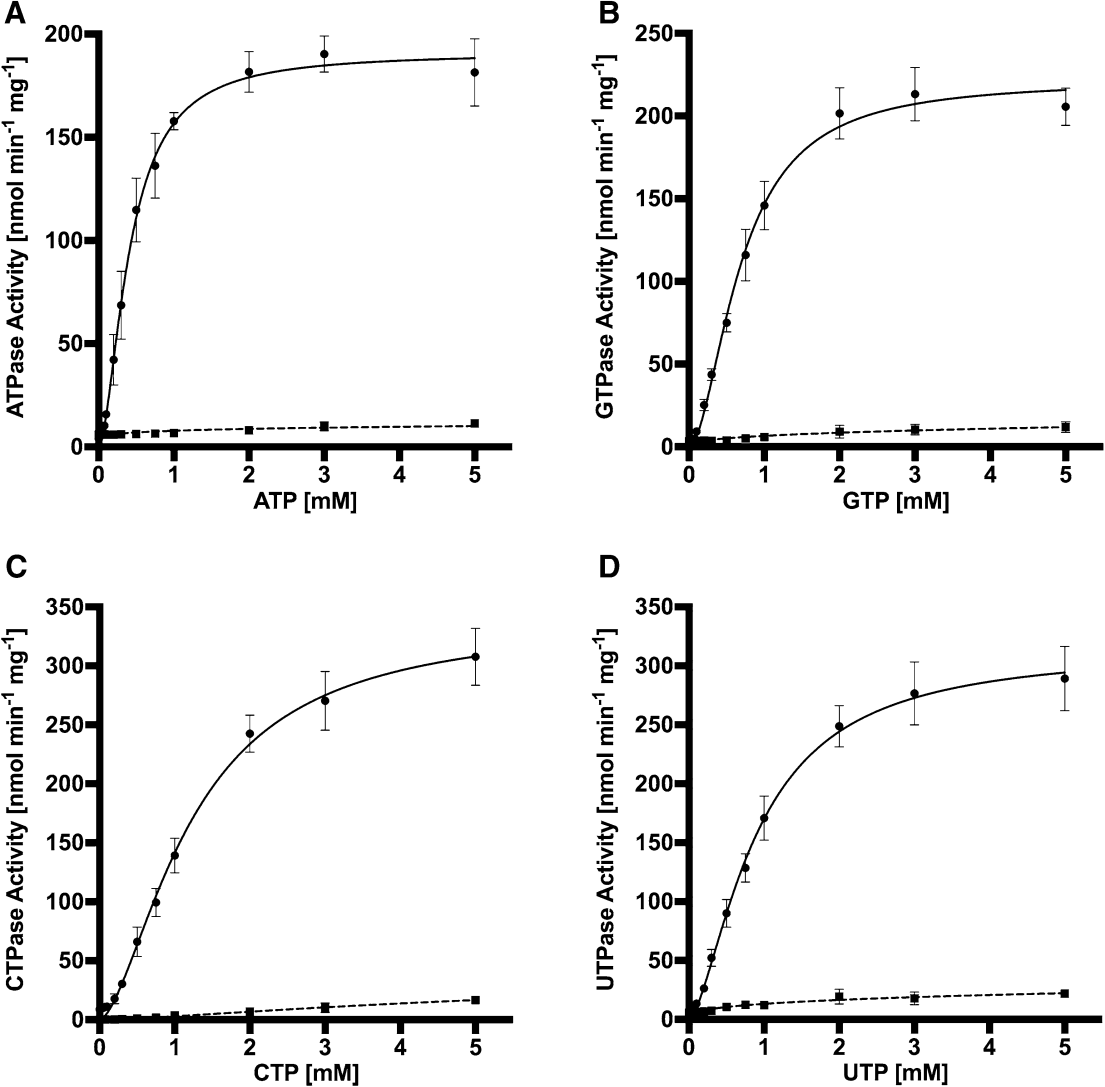
Kinetic measurement of *Sa*NsrF_WT_ (black) and *Sa*NsrF_H202A_ (dashed lines) NTPase Activity [nmol min^−1^ mg^−1^] after 18 min of incubation. A concentration range of each NTP from 0 to 5 mM was applied on freshly purified *Sa*NsrF or *Sa*NsrF_H202A_ (0.1 mg mL^−1^; diluted in 100 mM HEPES at pH 7). The reaction was stopped after 18 min and dyed for 7 min. A sigmoidal fit was applied using GraphPad PRISM 8.3.0. (**A**) Kinetic parameters of *Sa*NsrF_WT_ exposed to 0–5 mM ATP: v_max_: 190.9 ± 10.0 [nmol min^−1^ mg^−1^], h: 1.72 ± 0.27, k_half_: 0.41 ± 0.05 [mM]. (**B**) Kinetic parameters of *Sa*NsrF_WT_ exposed to 0–5 mM GTP: v_max_: 221.6 ± 11.1 [nmol min^−1^ mg^−1^], h: 1.82 ± 0.27, k_half_: 0.69 ± 0.07 [mM]. (**C**) Kinetic parameters of *Sa*NsrF_WT_ exposed to 0–5 mM CTP: v_max_: 339.0 ± 30.4 [nmol min^−1^ mg^−1^], h: 1.63 ± 0.53, k_half_: 1.23 ± 0.20 [mM]. (**D**) Kinetic parameters of *Sa*NsrF exposed to 0–5 mM UTP: v_max_: 314.8 ± 23.4 [nmol min^−1^ mg^−1^], h: 1.55 ± 0.25, k_half_: 0.90 ± 0.13 [mM]. All experiments have been performed in at least three biological replicates and are represented as means ± s.d.

As depicted in Fig. 2A, the *Sa*NsrF_WT_ protein demonstrated a nonlinear dependency of ATPase activity over a range of 0–5 mM ATP. The maximal reaction velocity was calculated to be 190.9 ± 10.0 nmol min^−1^ mg^−1^ when using ATP. Moreover, the calculation of the kinetic parameters resulted in a kinetic constant of k_half_ = 0.41 ± 0.05 mM and a Hill coefficient of h = 1.72 ± 0.27 (Fig. 2A and Table 1). A Hill coefficient > 1 demonstrates a cooperative behaviour, and suggests that *Sa*NsrF_WT_ needs to dimerize to hydrolyze ATP, which is in line with other previously characterized NBDs^41–43^. For GTP, the maximal reaction velocity was 221.6 ± 11.1 nmol min^−1^ mg^−1^ with a Hill coefficient of h = 1.82 ± 0.27 and a k_half_ value of 0.69 ± 0.07 mM (Fig. 2B and Table 1). Interestingly, the highest reaction velocity with a value of 339.0 ± 30.4 nmol min^−1^ mg^−1^ was reached using CTP as a substrate with the highest measured k_half_ value of 1.23 ± 0.20 mM and a Hill coefficient of 1.63 ± 0.53 (Fig. 2C and Table 1). The kinetic parameters using UTP as a substrate resulted in comparably high values of v_max_ = 314.8 ± 23.4 nmol min^−1^ mg^−1^, k_half_ = 0.90 ± 0.13 mM and h = 1.55 ± 0.25 (Fig. 2D and Table 1). The variant *Sa*NsrF_H202A_ displayed no hydrolytic activity for any of the four used NTPs (Fig. 2, dashed lines).

**Table 1:** Kinetic parameters V_max_ [nmol min^−1^ mg^−1^], k_half_ [mM] and the Hill-coefficient h resulting from different NTPs as a substrate for *Sa*NsrF_WT_.

NTP	V_max_	k_half_	h
ATP	190.9 ± 10.0	0.41 ± 0.05	1.72 ± 0.27
GTP	221.6 ± 11.1	0.69 ± 0.07	1.82 ± 0.27
CTP	339.0 ± 30.4	1.23 ± 0.20	1.63 ± 0.53
UTP	314.8 ± 23.4	0.90 ± 0.13	1.55 ± 0.25

All experiments have been performed in at least three biological replicates and are represented as means ± s.d.

### Structural models of SaNsrF monomer and dimer

Since no experimental structure of *Sa*NsrF is available, we generated a structural model of the NBD by comparative modeling. NBDs are the most conserved parts of ABC transporters and in the case of *Sa*NsrF, the templates used for modeling show a sequence identity of ~ 30–40% and a sequence similarity of 84–89% (Table S1). Of these X-ray structures (resolution between 1.7 and 3.4 Å), two constitute NBDs in the functionally active assembly; they were crystallized with the TMD of the macrolide exporter MacAB from *Acinetobacter baumannii* (PDB ID 5GKO^44^) and MacAB-like from *Streptococcus pneumoniae* (PDB ID 5XU1^45^).

**Figure 3:**
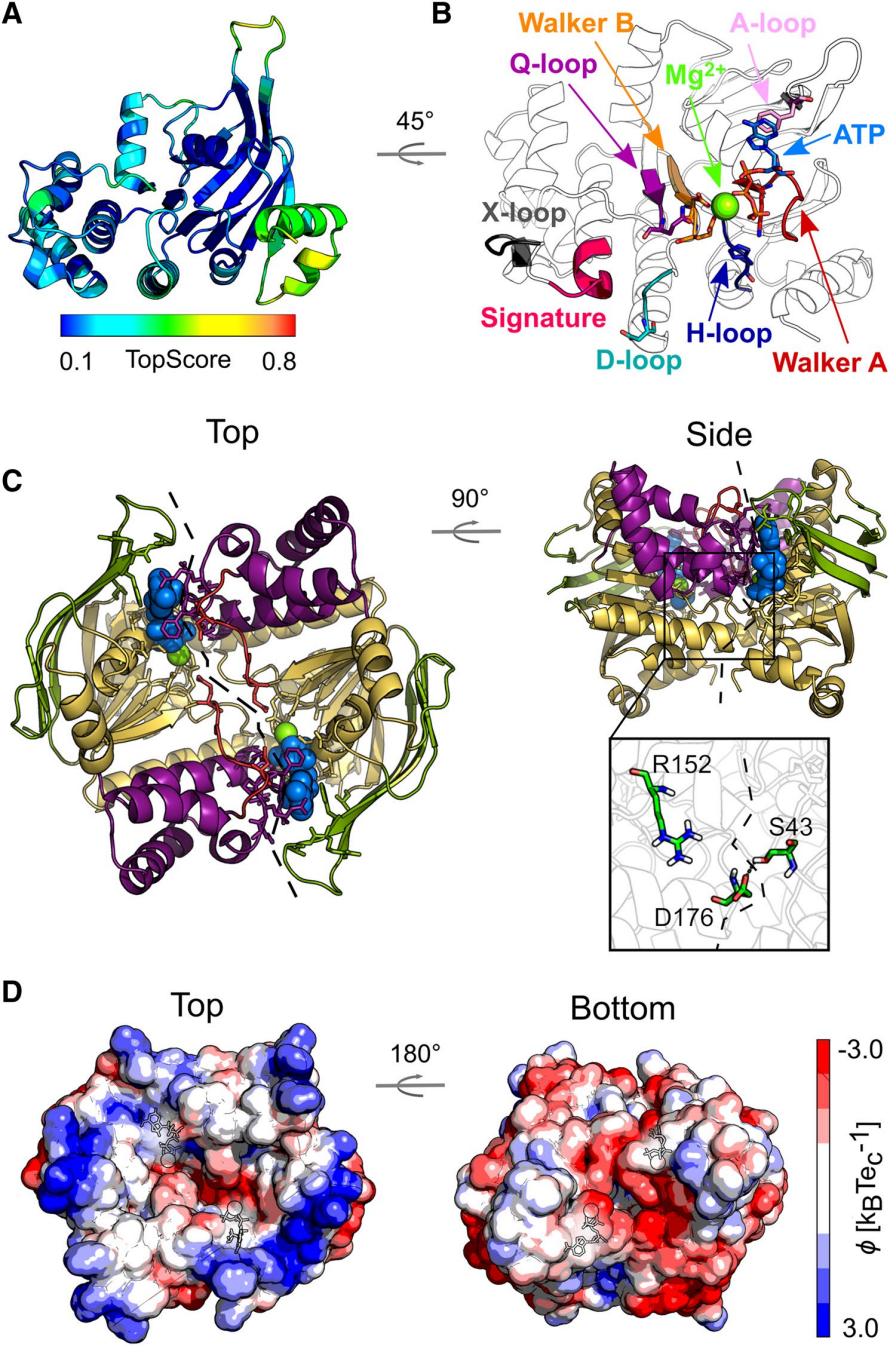
Homology models of *Sa*NsrF_WT_ monomer (**A**, **B**) and dimer (**C**, **D**). (**A**) Structure colored according to the residue-wise TopScore. Green/yellow colors indicate regions with low residue-wise error (< 50%). (**B**) Zoom into the NBD-NBD interface with ATP and Mg^2+^ bound, highlighting the conserved motifs necessary for ATP binding and hydrolysis, and for NBD-NBD and NBD-TM communication. See Table S2 for the location of the conserved motifs in the primary sequence^22^. (**C**) Structure colored according to domain organization and zoom into the NBD–NBD interface, reporting the conserved residues used as restraints for protein–protein docking. The α-helical domain is shown in violet; the RecA-like domain, further subdivided into F1-type ATP binding core, antiparallel β subdomain, and γ-phosphate linker is colored respectively in yellow, green, and red. The bound ATP (blue) and Mg^2+^ (green) are shown in space-filling representation. The dashed line highlights the interface between subunits. (**D**) Electrostatic potential computed for the representative structure of the most populated cluster of conformations obtained by MD simulations. The color scale of the electrostatic potential ranges from − 3.0 (red) to + 3.0 (blue) *k*_B_Te_c_^−1^; the potentials were computed with the Adaptive Poisson-Boltzmann Solver (APBS)^49^.

The homology model of *Sa*NsrF_WT_ in the monomeric form is of high quality, given the low overall TopScore^46^ (TS) value of 0.24 (Fig. 3A). This superimposition-free score evaluates local distance differences^47^ of all atoms in a model, and a value closer to zero indicates higher quality. The regions modeled with lower reliability (TS > 0.5), accounting only for ~ 6% of the total sequence, are located at the β-hairpin (residues 15–18) and the two C-terminal helices (residues 229–232, 235–236, 246–250). Both substructures can be found in other NDBs, however, indicating the plausibility of the model. For example, when compared to the structure of ComA from *Streptococcus mutans* (PDB ID 3VX4^48^), the C-terminal helices have a virtually identical fold, with an RMSD of 0.6 Å for the last 50 residues, based on sequence alignment followed by structural superimposition.

The dimeric *Sa*NsrF_WT_ model is structurally similar to other known structures, given RMSD values of ~ 5 Å or lower (RMSD of 3.5 Å, 4.5 Å and 5.2 Å for PDB IDs 1L2T, 5GKO, and 5XU1, respectively), indicating the suitability of the performed protein–protein docking. The reliability of the model is additionally verified by the presence of conserved motifs (Fig. 2B and Table S2), such as the phosphate-binding loop (P-loop or Walker A motif), the cofactor-chelating region (Walker B motif), and a short consensus sequence “LSGGQ” (C-loop or ABC signature motif), which signify ABC transporter family membership at the sequence level. Moreover, the α-helical and RecA-like domains are in the canonical head-to-tail arrangement (Fig. 3C). Interestingly, the calculated electrostatic potential shows a clear polarization (Fig. 3D) with positively charged residues (such as R and K) prevalent on the dimer’s side oriented towards the membrane (named “top”) and negatively charged residues (such as D and E) on the opposite side (named “bottom”) in agreement with the expected topology.

### Structural dynamics at the NBD–NBD interface and impact of the SaNsrF_H202A_ substitution

The *Sa*NsrF models were subjected to all-atom MD simulations of in total 10 μs length to investigate the structural dynamics at the NDB-NDB interface and to highlight the impact of the H202A substitution on ATP/Mg^2+^ binding. The RMSD profiles for *Sa*NsrF_WT_ and *Sa*NsrF_H202A_ monomers (Fig. S2) reach almost immediately a plateau at ~ 4 Å, indicating that the overall structure is mostly invariant over simulation times of 0.5 μs for each replica. Additionally, the low variability of ATP/Mg^2+^ coordinates (Fig. S3A,B) suggests that the *Sa*NsrF_H202A_ substitution does not impact ATP/Mg^2+^ binding, at least on the timescale of our simulations.

The RMSD profile for the *Sa*NsrF_WT_ and *Sa*NsrF_H202A_ dimers is mostly invariant (Fig. S4A) when the structures are superimposed onto the two subunits separately (red and blue lines). However, when the superimposition is done with respect to the least mobile regions in the whole dimer (black line), RMSD values reach ~ 6–9 Å in three out of five replicas for *Sa*NsrF_WT_, indicating that the arrangement of the two subunits changes during the simulations. In particular, the interface between the subunits partially opens (Fig. S4B) up to ~ 25 Å (Fig. S5). The change of ATP molecule and Mg^2+^ ion positions relative to the protein is more marked for *Sa*NsrF_WT_ dimer (Fig. S3). Interestingly, this is not happening in the *Sa*NsrF_H202A_ variant, where the interface seems to be more stable.

**Figure 4:**
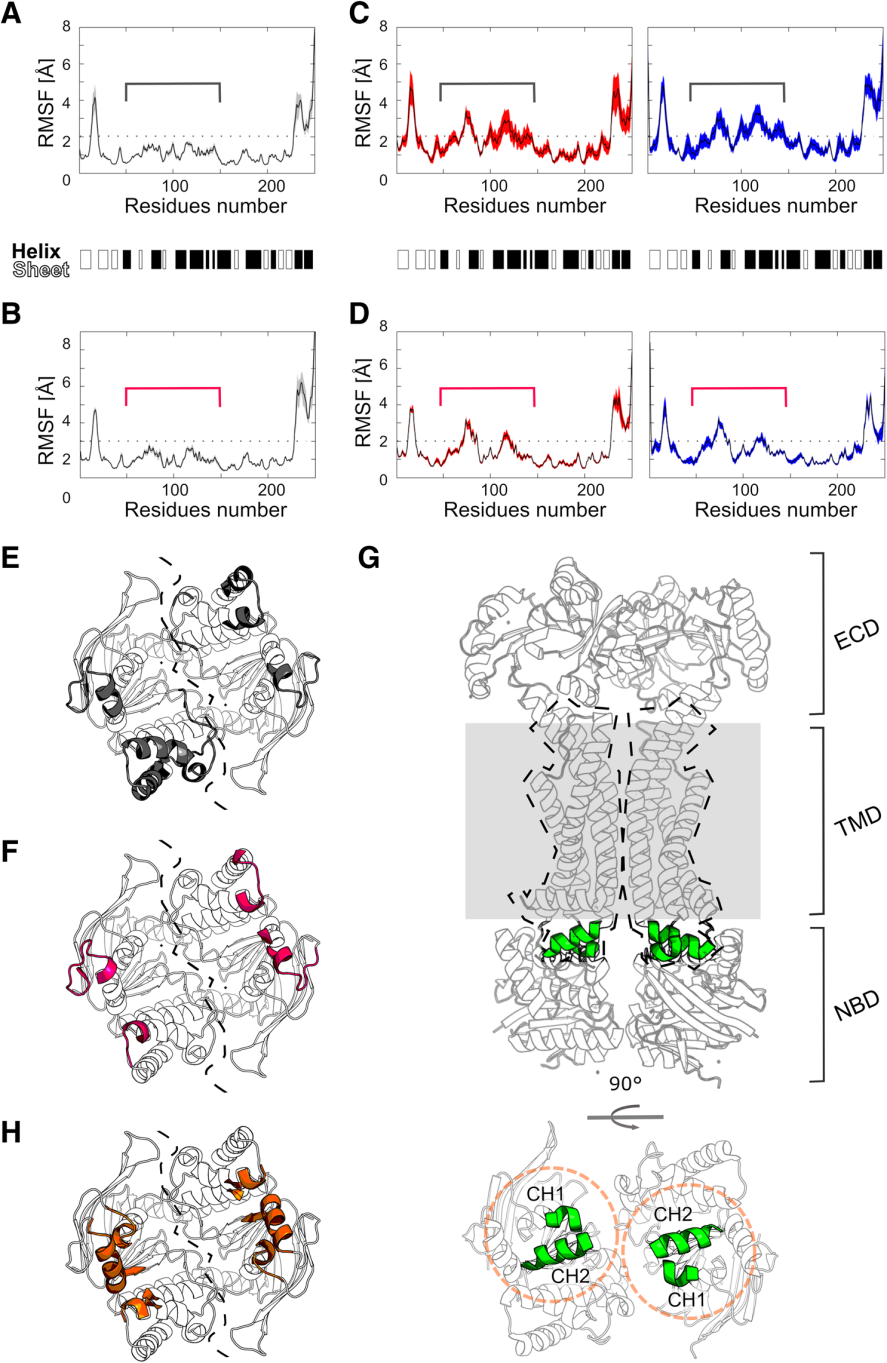
Structural mobility of the *Sa*NsrF_WT_ and *Sa*NsrF_H202A_ systems expressed as RMSF of Cα atoms. Before RMSF calculation, the structures were fitted onto the 15% least mobile residues, averaged over five MD simulation replicas. The variability between replicas is expressed as SEM and shown as colored area (grey for the monomers, red for chain A and blue for chain B). (**A**) *Sa*NsrF_WT_ monomer. (**B**) *Sa*NsrF_H202_ variant monomer. (**C**) *Sa*NsrF_WT_ dimer. (**D**) *Sa*NsrF_H202_ variant dimer. The secondary structure elements of the initial model are shown as black and white bands. The central region of *Sa*NsrF_H202_ (residues ~ 50–150) is highlighted with brackets. Residues of the central region with RMSF > 2 Å are mapped onto the dimer structures. (**E**) For *Sa*NsrF_WT_ (in grey) and (**F**) for *Sa*NsrF_H202_ variant (in pink). The other two regions with RMSF > 2 Å (hairpin of the antiparallel β subdomain and the C-term) are not shown for clarity. The dashed line highlights the interface between subunits. (**G**) Structure of the MacAB-like transporter from *Streptococcus pneumoniae* (PDB ID 5XU1^45^) reported as comparison to highlight the expected orientation of the NBD to the TMD (shown as dashed shape), its coupling helices (CH1 and CH2, highlighted in green) and the membrane (as grey area). (**H**) After superimposition of the NBDs, regions of *Sa*NsrF located at < 5 Å from the coupling helices of the MacAB-like structure, and therefore likely involved in NBD-TMD communication, are highlighted in orange.

In terms of structural mobility, the central region of *Sa*NsrF_WT_ and *Sa*NsrF_H202A_ (residues ~ 50–150) shows a different profile in monomers and dimers (Fig. 4). In monomers (Fig. 4A,B), this region is less mobile than in dimers (Fig. 4C,D), with RMSF values lower than 2 Å and up to 4 Å, respectively. Moreover, in the dimeric *Sa*NsrF_H202A_ variant, this region is slightly less mobile than in *Sa*NsrF_WT_. The residues of the central region are oriented towards the TM region of the transporter (Fig. 4E,D). In addition, after the alignment of *Sa*NsrF with NBDs of structures containing the TMD (PDB ID 5XU1, Fig. 4G), most of the residues of this central region are located at < 5 Å distance from the coupling helices (CH1, between TM2 and TM3, and C-terminal CH2) of the transporter, suggesting that this central region is involved in NBD-TMD communication (Fig. 4H). A similar result was found for the HlyB transporter^50^, where the X-loop motif (corresponding to residues 137–142 in *Sa*NsrF, located in the central region) has been proposed to be an important part of the NBD-TMD communication. Even though we are considering an ATP-bound pre-hydrolysis state, *Sa*NsrF in the dimer seems to be generally more mobile than in the monomer, in agreement with the idea that a dimeric assembly is needed in order to perform its function.

**Figure 5:**
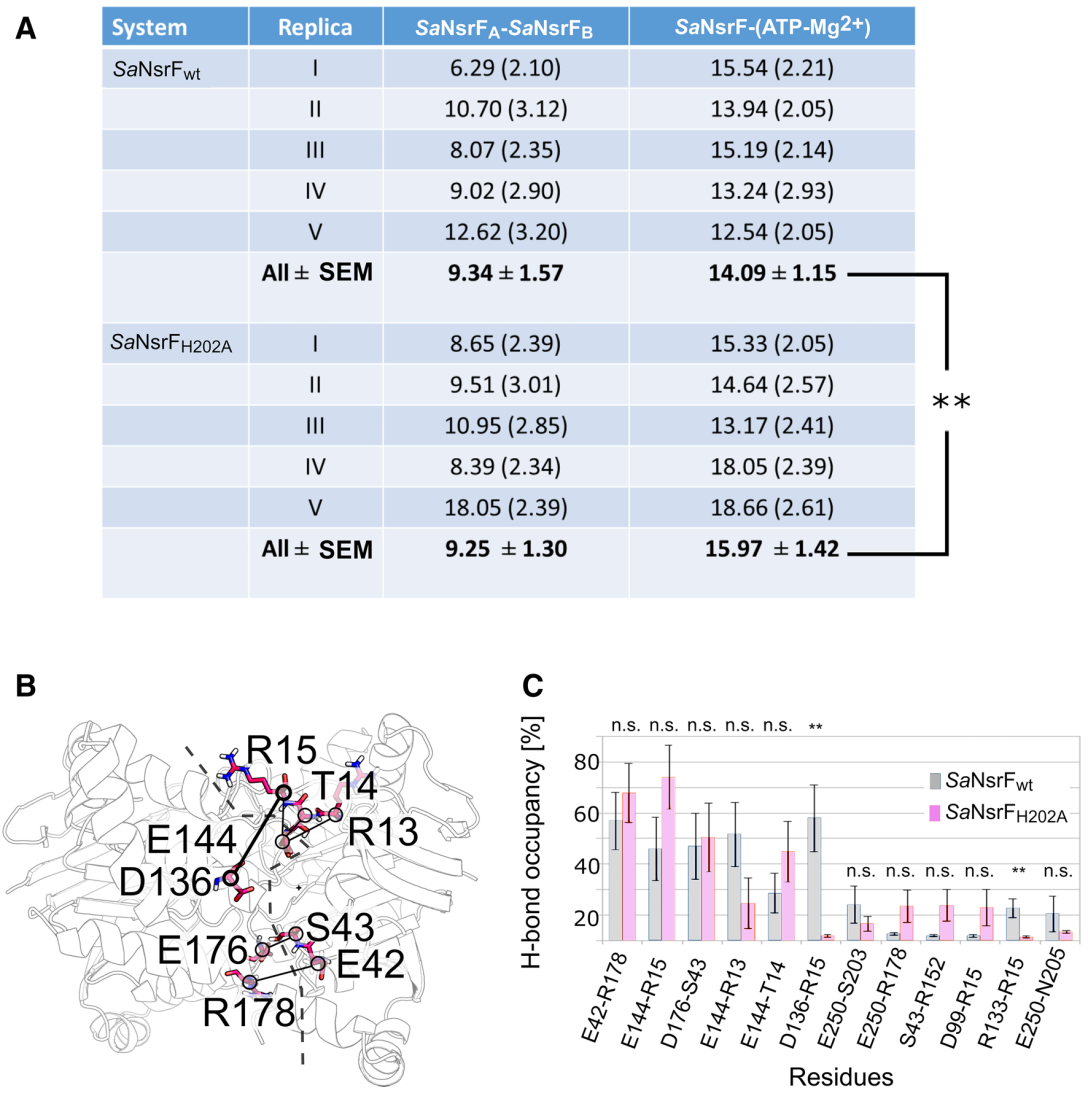
H-bond analysis in *Sa*NsrF_WT_ and *Sa*NsrF_H202A_ dimers. (**A**) The average number of H-bonds between the two proteins and between the protein and the ligands per MD replica. Standard deviations are reported in parentheses. For the numbers in bold, the SEM was computed according to *n* = 5. **p < 0.01 according to a two-tailed *t*-test. (**B**) Residues in the interface that predominantly form H-bonds (occupancy > 20%). H-bonds are shown as lines connecting the Cα atoms of these residues. The dashed line highlights the interface between subunits. (**C**) H-bond occupancy for the most prevalent interactions (occupancy in at least one of the systems > 10%). Error bars are showing the SEM. **p < 0.01 according to a two-tailed *t*-test for the comparison of *Sa*NsrF_WT_ and *Sa*NsrF_H202A_ variant; n.s.: not significant.

H-bond analysis in *Sa*NsrF_WT_ and *Sa*NsrF_H202A_ dimers reveals that the number of H-bond interactions between *Sa*NsrF and the ligands (ATP molecules and magnesium ions) is on average higher in the case of the *Sa*NsrF_H202A_ variant (Fig. 5A). This is due to the higher structural stability compared to *Sa*NsrF_WT_. Besides the three residues used as restraints for protein–protein docking (S43–R152–D176), other residues contribute to the stability of the dimer with H-bond occupancies up to 70%, such as R13, T14, R15, E42, E144, and R178 (Fig. 5B,C). Surprisingly, the residue-wise H-bond occupancy in *Sa*NsrF_WT_ is significantly higher (*p* < 0.01) for two specific H-bonds involving both side chains and backbone atoms (D136–R15 and R133–R15), although the interface of the *Sa*NsrF_WT_ dimer is less structurally stable (see above). Indeed, in the initial dimeric model, these interactions are not present, but require the movement of one monomer to the other for them to form.

To conclude, the generated models show a high structural stability over the simulation lengths. In the dimers, the central region is more mobile than in the monomers; in *Sa*NsrF_WT_, the interface between subunits is structurally less stable than in the *Sa*NsrF_H202A_ variant. Since a shift of one monomer to the other is necessary for NDBs to perform their function, these results together suggest that the mutation *Sa*NsrF_H202A_ impacts the structural dynamics at the *Sa*NsrF interface and not only the catalytic mechanism.

### Small angle X-ray scattering

**Figure 6:**
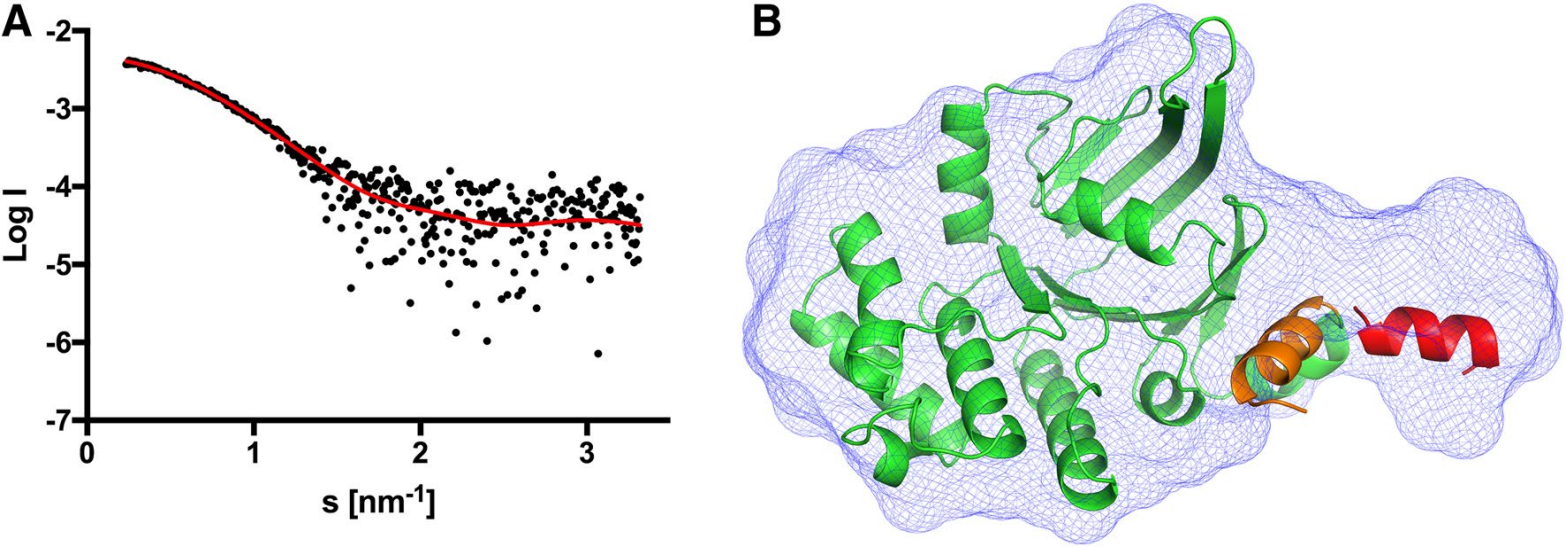
Comparison of the ab initio model with the homology model. (**A**) Experimental scattering data are shown as black dots and the ab initio model fit as red line. The intensity is displayed as a function of momentum transfer *s*. (**B**) Ab initio model of the *Sa*NsrF_WT_. The volumetric envelope from *Sa*NsrF_WT_, calculated from the scattering data using GASBOR^51^, is shown by the blue mesh. The homology model of the *Sa*NsrF_WT_ monomer (shown in green) was docked into the volumetric envelope using SUPCOMB^56^. Concerning the flexibility of the C-terminal helix (shown in orange), we show a possible, changed orientation of this helix in red.

Unfortunately, we were not able to crystalize the *Sa*NsrF protein, although extensively tried. In order to experimentally validate this new model, we choose Small Angle X-Ray Scattering (SAXS) to compare the theoretical model with the experimental scattering (Fig. 6A) measured with the Xenocs Xeuss 2. Based on the experimental data, we calculated an ab initio model for *Sa*NsrF_WT_ with the program GASBOR^51^ and obtained a χ^2^ value of 0.97. Superimposing the ab initio and the TopModel model reveals that the structure and the envelope obtained by the SAXS experiment overlap, but also a density tail at the C-terminus of *Sa*NsrF_WT_ (Fig. 6B) that is not occupied by the model. Scrutinizing the templates used by TopModel^52^ shows that this helical part (Fig. 6B, orange helix) is rather unstructured or even missing. This finding indicated that this region might be highly flexible in solution, thereby covering the available free space in the SAXS envelope (Fig. 6B, red helix). With the program CRYSOL^53^ we compared the theoretical scattering curve obtained from the TopModel model against the experimental data. The resulting χ^2^ value of 1.16 indicates a good agreement between the prediction and the experiment. We uploaded the SAXS data and the corresponding model of *Sa*NsrF to the Small Angle Scattering Biological Data Bank (SASBDB)^54,55^ with the accession code SASDJR3.

### Molecular docking of other NTPs

**Figure 7:**
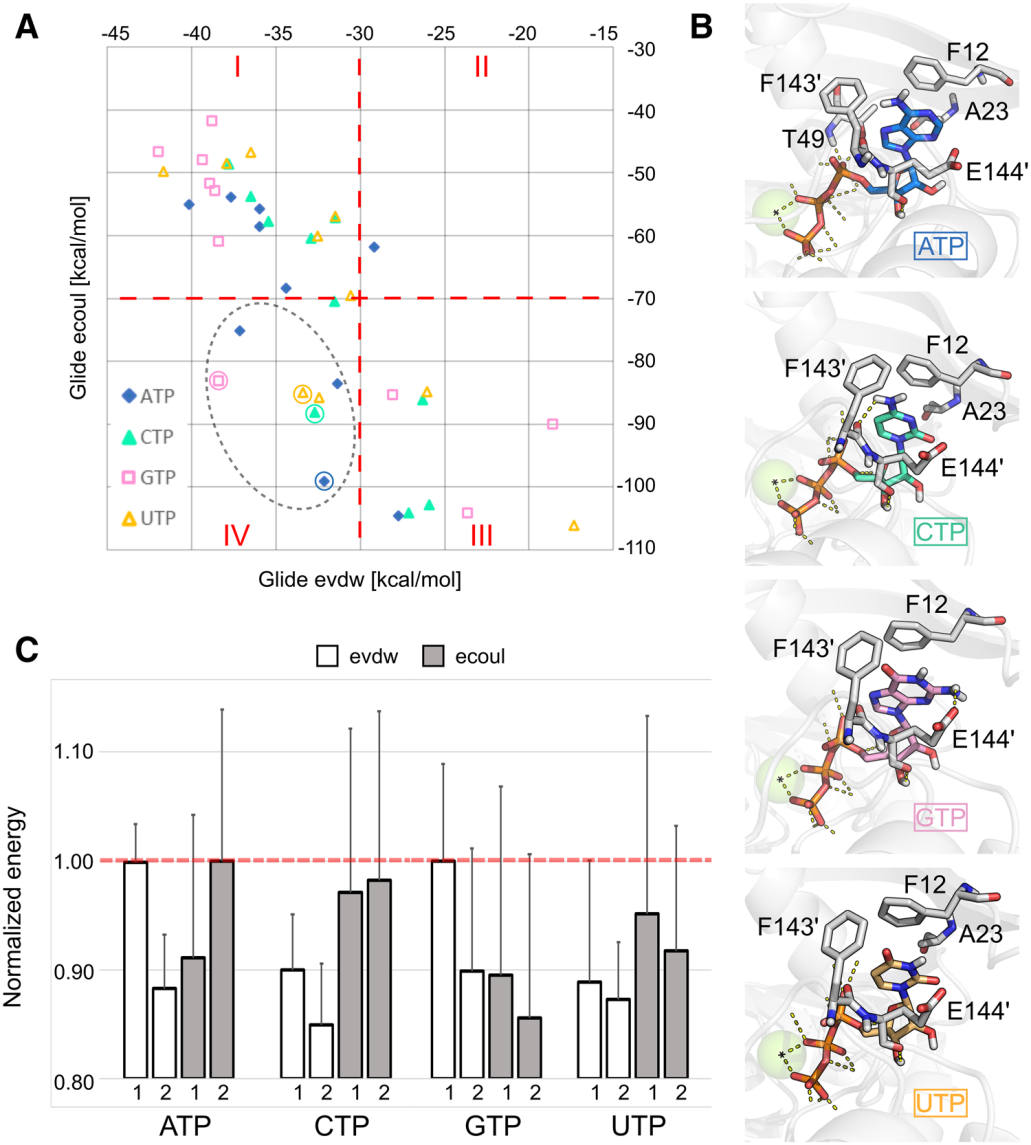
Molecular docking of other NTPs. (**A**) Scatterplot representing the Coulomb (ecoul) versus the van der Waals (evdw) energy terms of the docking score. Each data point represents an NTP configuration inside the two pockets of five different, equilibrated *Sa*NsrF structures. In quadrant IV, NTP configurations with respective lowest energies are circled. (**B**) Representative binding modes of NTPs, referring to the circled data points in section A. Residues at ≤ 4 Å from the nucleobases are shown in sticks and labelled. The Mg^2+^ ion is shown as a green sphere (**C**) Normalized average energy terms for pockets 1 and 2 of each SaNsrF complex. The error is reported as normalized SEM (*n* = 5).

In order to rationalize the hydrolysis preference for ATP over other NTPs, we predicted the binding mode of these molecules in complex with the *Sa*NsrF_WT_ dimer. Ten different pocket conformations, obtained from five equilibrated structures used also for MD simulations times two pockets each, were considered. When focusing on the configurations with lowest Coulomb (ecoul) and van der Waals (evdw) energies, ATP is slightly enriched compared to the other NTP (3 × ATP, 2 × UTP, 1 × CTP and 1 × GTP), suggesting that ATP binding is preferred due to enthalpic contributions to binding (Fig. 7A). Residues giving rise to this preference are those interacting with the nucleobase, namely F12, T49, A23 of one subunit and F143′ and E144′ of the other (Fig. 7B). In particular, the phenylalanines are interacting with the nucleobase by π-π stacking interactions, and the amino groups of CTP and GTP form H-bonds with the backbone oxygen of F143′ and the carboxylate group of E144′, respectively. Since in ATP the amino group has the same orientation as in CTP, a similar kind of H-bond pattern can be expected.

Over respective pockets 1 or 2, which are not symmetric as described above, ATP shows the largest sums of Coulomb and van der Waals energies compared to the other NTPs (Fig. 7C), indicating strongest binding based on enthalpic components, which is in line with the biochemical data where ATP shows the lowest k_half_ value (Fig. 2 and Table 1).

## Discussion

A rather novel family of ABC-transporters, the Bacitracin efflux (Bce) type transporters, have been identified to confer high-level resistance against bacitracin as well as against lantibiotics such as nisin and gallidermin in *Bacillus subtilis, Staphylococcus aureus,* and *Streptococcus agalactiae*^8,14,16,57–60^. These transporters have been rudimentarily characterized in vitro*.* We set out to characterize the NBD of the transporter *Sa*NsrFP; this transporter has been shown to be involved in lantibiotic resistance^8^.

We have purified and characterized the *Sa*NsrF_WT_ and *Sa*NsrF_H202A_ proteins regarding their ability of ATP hydrolysis. The results revealed that inorganic phosphate is only released in a pH range of 6–8, where an HEPES buffer at pH 7 was found to yield maximal ATPase activity. Interestingly, 20% difference could be found in a TRIS buffer system at the same pH (Fig S1A). Similar results were obtained by Zaitseva et al. examining the HlyB-NBD^36^. In that study, a correlation between the pH of 6 and the pK_a_ values of the glutamate residue and/or the γ-phosphate of the nucleotide and between the pH of 8 and the pK_a_ value of the conserved histidine bound in a salt bridge with the γ-phosphate was made. On that basis, the nucleophilic attack on the γ-phosphate is preceded, originating from a hydrolytic water molecule, which results in the cleavage of the γ-phosphate moiety^26,36,61^. Moreover, the importance of the conserved histidine could be confirmed since the *Sa*NsrF_H202A_ variant was shown to be incapable of hydrolysing ATP. Here, the ‘linchpin’-role during ATP-hydrolysis is conducted by the H-loop^22,36,38,62^. Also, this allows a possible explanation for the observed decrease of activity with increasing concentrations of NaCl (Fig. S1B). Since the conserved histidine is in contact with the γ-phosphate of the nucleotide by forming a salt bridge, rising salt concentration could disrupt this existing interaction. In contrast, a buffer system containing 300 mM of NaCl was used for protein storage, which indicates an inverse correlation between protein stability and activity at rising NaCl concentrations^63^. The incapability of *Sa*NsrF_H202A_ to hydrolyse ATP supports in vivo studies where a loss of resistance against the lantibiotic nisin was observed when expressed in *L. lactis* bacterial cells^8^.

Like many other NBDs, *Sa*NsrF was observed to be strictly dependent on its cofactor Mg^2+^^39,64,65^, because this is required as a Lewis acid in the catalytic cycle. Mg^2+^ is involved in proton abstraction from the nucleotide and the nucleophilic attack of the catalytic water, which results in the hydrolytic cleavage of its γ-phosphate^36^.

Finally, we conducted kinetic measurements including all optimized parameters and the preference of *Sa*NsrF_WT_ and *Sa*NsrF_H202A_ for hydrolysing different NTPs. We propose that the main interaction of the nucleoside triphosphate and the protein occurs by π–π-stacking between the adenine moiety and F12 downstream of the Walker A motif (Fig. 3B,C) as also observed for other NBD’s^22,24,25,30^. Also, Mg^2+^, anchored to the protein through Asp and Glu residues of the Walker B motif, interacts with the phosphate region of ATP. The Walker A motif binds to the other side of the phosphate region (Fig. 3B).

Based on a comparison of docked binding poses of other NTPs, additional interacting residues were predicted (Fig. 7B). Amino group-containing NTPs (ATP, CTP and GTP) can form H-bonds with the backbone oxygen of F143′ and the carboxylate group of E144′, whereas purines in ATP and GTP form more extended π–π stacking interactions with F12 and F143′. ATP shows the largest sums of Coulomb and van der Waals energies compared to the other NTPs in either pocket of the NBD, in line with the biochemical data where ATP displayed the lowest k_half_ value (Fig. 2 and Table 1).

By comparing the measured kinetic parameters of each examined NTP, it becomes obvious that the reactions including UTP or CTP resulted in a significantly higher reaction velocity, respectively, when compared to ATP. Nevertheless, the CTPase and UTPase activities revealed noticeably high kinetic constants (k_half_) as well. With regards to the substrate affinity represented by the k_half_ value, a minimum of 0.41 ± 0.05 mM was reached using ATP as a substrate, which signifies ATP as the most favoured of all four tested NTPs for *Sa*NsrF_WT_. Hence, ATP has the highest affinity to *Sa*NsrF_WT_ compared to the other examined NTPs, which corresponds with the physiological appearance in vivo of each NTP ([ATP] > [GTP] > [UTP] > [CTP]), which underlines that ATP is the preferred substrate for the protein^32,66–68^. Considering the physiology of purine (ATP, GTP) and pyrimidine (UTP, CTP) nucleotides, we concluded that the involved aromatic ring systems play a major role concerning the substrate affinity and stability of the protein-substrate-complex. Here, pyrimidine bases exhibit a smaller electron density that can be involved in π–π-stacking. Thus, dissociation of pyrimidine nucleotides from the enzyme occurs faster than purine nucleotides. By contrast, the stabilized protein–purine-complex is less liable to dissociation. Together, this may explain the small k_half_ values found for ATP and GTP and the high reaction velocities caused by a high turnover of CTP and UTP.

NBDs are assumed to share a large number of properties due to highly conserved sequences and specific motifs (see Fig. 3B,C and Table S2)^22–26,30^. The presence of a certain substrate such as ATP is supposed to induce a dimerization of the two NBD monomers in a typical head-to-tail formation, resulting in two ATP molecules in the dimer interface, sandwiched by the Walker A motif of one monomer and the signature motif of the other one as a cooperative process^22,24,25^.

NBDs hydrolyse ATP, which drives substrate translocation by conformational changes of the TMD. In the case of the BceAB-type ABC transporter *Sa*NsrFP, the energy supply is provided by the BceA-domain *Sa*NsrF^16^. By employing SEC-MALS-coupled analysis we were able to confirm a monomeric state of *Sa*NsrF_WT_ and its variant *Sa*NsrF_H202A_ in solution since the measured molecular masses corresponded with the calculated values for each monomer. This agrees with the oligomeric state of other NBDs from other ABC transporter families in the absence of nucleotide^34–36^.

Furthermore, this is in line with our SAXS data that allowed the construction of a volumetric envelope of the *Sa*NsrF_WT_ monomer. The experimental structure of *Sa*NsrF has not been published yet. Here, we generated a structural model using TopModel^52^ based on five main templates 1F3O_A, 5XU1_B, 2PCL_A, 5GKO_A, 2OLJ_A (Fig. 3A, 6B). We compared this model with the volumetric envelope obtained from SAXS data, showing high reliability and agreement with experimental data. It is striking that the density of the protein model is partly not occupied. A flexible C-terminus could be the reason, which would make a temporary fit of the versatile C-terminal helix to the proposed model possible. As for well-studied NBDs such as HisP, the modeled *Sa*NsrF dimer exhibits the typical head-to-tail formation including two sandwiched ATP molecules in the dimer interface between the Walker A motif of the first monomer and the C-loop of the second one^22,24,25,30^. Therefore, the *Sa*NsrF protein shares many structural similarities with other known NBDs. As the γ-phosphate moiety of ATP was predicted to be in close proximity of the conserved histidine (H-loop) and the cofactor Mg^2+^, one can deduce a consensus with the hypothesis of the H-loop acting as a sensor, whereas the cofactor is involved in hydrolytic cleavage while being coordinated by the Walker B motif (Fig. 3B,C)^22,23,26,28^. Furthermore, in *Sa*NsrF_WT_, the interface between subunits is structurally less stable than in *Sa*NsrF_H202A_. Since a shift of one monomer to the other is necessary for NDBs to perform their function, these results suggest that the substitution *Sa*NsrF_H202A_ impacts the structural dynamics at the *Sa*NsrF interface and not only the catalytic mechanism.

Clearly, the *Sa*NsrF protein represent an isolated NBD and we do not know if the kinetic correspond to the ATP hydrolysis that will occur in the presence of the transmembrane protein *Sa*NsrP. However, when comparing the data with known NBDs which has been described before in the presence and absence of the transmembrane segment it can be observed that v_max_ might be changed, the k_m_ values however remains very similar. For example the ATP hydrolysis kinetics have been described for the HlyB NBD as well as for the purified full length transporter in detergent solution^26,36,38,43,69^. Here the NBD showed a v_max_ of 200 nmol min^−1^ mg^−1^ with a k_m_ value of 0.31 where as the full length transporter displayed a lower v_max_ of 8.1 nmol min^−1^ mg^−1^ with a k_m_ value of 0.36. This reduction is likely due to the detergent, which is present to keep the HlyB transporter in solution. Important, however is that in both cases the kinetic displayed cooperativity (Hill coefficient > 1) as in the case of *Sa*NsrF and the corresponding histidine mutation also resulted in an inactive protein. This shows that our NTP analysis of the *Sa*NsrF will likely be similar even when the TMD *Sa*NsrP is present. The same observations were found for the nisin transporter NisT from *L. lactis*^70^ and the nukacin ISK-1 transporter NukT from *Staphylococcus arneri* ISK-1^71^ albeit in detergent solution.

In summary, the experiments revealed the first detailed insights into biochemical properties of the BceA domain of the BceAB-type ABC transporter *Sa*NsrFP. We showed that *Sa*NsrF_WT_ and its variant *Sa*NsrF_H202A_ exist as monomers in solution and determined several physiological and structural properties of the protein by evaluating its ATPase activity in comprehensive in vitro studies and molecular modelling and simulations. Hence, this study contributes to the mechanistic and structural understanding of the BceAB-type ABC transporter family, which opens up the possibility to pharmacologically target this family in order to combat multidrug-resistant species in the long run. It further confirms in vivo data where the H202A variant of *Sa*NsrF displayed a loss in the activity, which now can be pinpointed to a lack of ATP hydrolysis, and shows that this variant can well serve as a negative control in studies concerning BceAB type transporters since the histidine is conserved throughout the sequence of this family.

## Materials and methods

### Expression of SaNsrF_WT_ and SaNsrF_H202A_

*E. coli* BL21 (DE3) strains were transformed via heat shock method^72^ with pET-16b-NHis_10_-*Sa*NsrF_WT_ or pET-16b-NHis_10_-*Sa*NsrF_H202A_, respectively. Precultures were selectively grown with 20 µg mL^−1^ ampicillin at 37 °C and 180 rpm overnight. Lysogeny Broth (LB) medium was pre-incubated with 20 µg mL^−1^ ampicillin and inoculated with the respective preculture to an OD_600_ of 0.1. The cultures were grown to an OD_600_ of 0.4 at 37 °C and 180 rpm whereupon the temperature was reduced to 18 °C. Protein expression was induced by the addition of 1 mM IPTG at an OD_600_ of 0.8 and the cultures were further grown overnight.

### Protein purification

*Sa*NsrF_WT_ and *Sa*NsrF_H202A_ were purified using Immobilized Metal Ion Chromatography (IMAC). Therefore, a 5 mL HiTrap Chelating HP column, loaded with Zn^2+^, was equilibrated with low IMAC-buffer (100 mM HEPES at pH 8, 300 mM NaCl, 20% glycerol). Protein elution was undertaken with the high IMAC-buffer (low IMAC-buffer plus 125 mM histidine). A washing step of 40-percent high IMAC-buffer was introduced before. The concentrated eluted proteins were then injected onto a Superdex 75 16/60 size exclusion column at a flow rate of 0.5 mL min^−1^, pre-equilibrated with SEC buffer (100 mM HEPES at pH 8, 300 mM NaCl, 20% glycerol). Protein eluates were collected and stored at 4 °C.

### ATPase activity assay

The ATPase activity of *Sa*NsrF_WT_ and *Sa*NsrF_H202A_ (diluted in 100 mM HEPES at pH 8, 100 mM NaCl) was examined by the Malachite Green Phosphate Assay at a protein concentration of 0.1 mg mL^−1^ that was initially undertaken at room temperature (20 °C). Several parameters were screened to determine the optimal buffer and temperature conditions for the protein activity (see Supplementary Information).

Kinetic measurements for *Sa*NsrF_WT_ and *Sa*NsrF_H202A_ were performed under the influence of NTP (ATP, GTP, CTP, UTP) with concentrations ranging from 0 to 5 mM.

Therefore, the kinetics were fitted using the Hill equation:

$$ Y = \frac{{v_{max} \times X^{h} }}{{\left( {k_{half}^{h} + X^{h} } \right)}}. $$

Y: ATPase activity [nmol min^−1^ mg^−1^], X: substrate concentration [mM], k_half_: substrate concentration at half-maximal reaction velocity [mM], h: Hill coefficient.

All shown data are representing the average of a triple evaluation at least, with the standard deviation reported as errors.

### Small angle X-ray scattering (SAXS)

We collected all SAXS data on our Xeuss 2.0 Q-Xoom system from Xenocs, equipped with a PILATUS 3 R 300 K detector (Dectris) and a GENIX 3D CU Ultra Low Divergence x-ray beam delivery system (Xenocs). The chosen sample to detector distance for the experiment was 0.55 m, results in an achievable q-range of 0.18–6 nm^−1^. All measurements were performed at 15 °C with protein concentrations between 0.5 and 4.2 mg mL^−1^. Samples were injected in the Low Noise Flow Cell (Xenocs) via autosampler. For each sample, twelve frames with an exposer time of ten minutes were collected. By comparing these frames, we excluded the possibility of aggregation and radiation damage during the measurement. Data were scaled to absolute intensity against water. All used programs for data processing were part of the ATSAS Software package (Version 3.0.1), available on the EMBL website^73^. Primary data reduction was performed with the program PRIMUS^74^. With the Guinier approximation we determined the forward scattering *I*(*0*) and the radius of gyration (*Rg*)^75^. The program GNOM was used to estimate the maximum particle dimension (*D*_*max*_) with the pair distribution function *p*(*r*)^76^. Low resolution ab initio models were calculated using GASBOR^51^. The superposition of a predicted *Sa*NsrF model (see below) was done using the program SUPCOMB^56^.

### Structural models of SaNsrF complexes

As an experimental *Sa*NsrF structure is not available, a homology model was constructed using the template-based protein structure prediction program TopModel^52^ and the *Sa*NsrF_WT_ sequence as input (NCBI Reference Sequence: WP_000923535.1). In order to build a *Sa*NsrF model arranged in a dimeric assembly with substrate (ATP) and cofactor (Mg^2+^) bound, starting from the *Sa*NsrF_WT_ monomer in the *apo* state, a search for sequence similarity and structural properties was performed on the Protein Data Bank. The results were filtered according to the following criteria: sequence identity ≥ 33% and E-value cutoff 0.001 as determined by BLAST^77^; oligomeric state equals 2; sequence length of 250 ± 50 residues; resolution ≤ 2 Å. Out of six results, only one (PDB ID: 1L2T^28^) is crystallized as a functionally active “ATP sandwich” symmetrical dimer and was therefore used as a reference. Since ATP is bound at the interface of the dimer and its binding is influenced by both protein subunits, both protein–ligand and protein–protein docking would be particularly challenging in this case. Hence, we constructed first the *Sa*NsrF_WT_ dimer in the *apo* form and the ATP/Mg^2+^-bound form subsequently.

To do so, protein–protein docking was performed with the program HADDOCK^78,79^, using distances between respective three residues that bridge the two subunits together with H-bond interactions as restraints (S40/S43, R153/R152 and D177/D176, for PDB ID 1L2T/*Sa*NsrF_WT_ sequences, respectively). The most similar docking solution to the reference PDB ID 1L2T was used for further modeling steps.

Both, *Sa*NsrF_WT_ monomer and dimer structures were preprocessed with the Protein Preparation Wizard^80^ of Schrödinger’s Maestro Suite. Since the residues at the binding sites are highly conserved, ATP and Mg^2+^ are considered to bind in a very similar way as in PDB ID 1L2T. Thus, their coordinates were copied from the reference into the *Sa*NsrF_WT_ model after alignment to one protein subunit. Residues located ≤ 5 Å away from the ATP molecules were energy-minimized using the OPLS 2005 force field^81^ with standard cutoff values for van der Waals, electrostatic, and H-bond interactions, until the average RMSD of non-hydrogen atoms reaches 0.30 Å. Bond orders as well as missing hydrogen atoms were assigned, and the H-bond network was optimized. Finally, residue 202 was substituted to construct the *Sa*NsrF_H202A_ variant of the monomer and dimer.

### Molecular dynamics simulations

In order to validate the modeled protein–protein interface and the ATP binding mode, and to investigate the impact of the *Sa*NsrF_H202A_ substitution on structural dynamics, a set of MD simulations was performed using Amber 2019^82^. Four different ATP/Mg^2+^-bound *Sa*NsrF systems were prepared for this with the LEaP program^83^: monomer and dimer, both for *Sa*NsrF_WT_ and *Sa*NsrF_H202A_.

After establishing charge neutrality by adding sodium counter ions, each system was placed in a truncated octahedral box of TIP3P^84^ water with a distance of the nearest atom to the border of the box of ≥ 11 Å. Structural relaxation, thermalization, and production runs of MD simulations were conducted with pmemd.cuda^85^ using the ff14SB force field^86^ for the protein, Joung-Cheatham parameters^87^ for ions, and available ATP parameters^88^. For each starting complex, five independent replicas of 500 ns length each were performed, resulting in a cumulative simulation time of 10 µs. In order to set up independent replicas and obtain slightly different starting structures, the target temperature was set to different values during thermalization (299.8 K, 299.9 K, 300.0 K, 300.1 K, 300.2 K and 300.3 K). A detailed description of the thermalization protocol can be found elsewhere^89^. The analysis of the MD trajectories was carried out with cpptraj^90^ on snapshots extracted every 1 ns. All the MD-generated conformations were clustered applying a hierarchical agglomerative approach and an RMSD cutoff value of 4 Å. The representative structure of the *Sa*NsrF_WT_ monomer was compared to the experimentally determined SAXS density.

The representative structure of the most populated cluster for the *Sa*NsrF_WT_ dimer was used to calculate the electrostatic potential with the Adaptive Poisson-Boltzmann Solver (APBS) software package^49^ as implemented in PyMOL^91^. Dielectric constants (*ε*) of 2.0 and 78.0 were used, respectively, for the protein and for water, and the concentration of monovalent cations and anions was set to 0.15 M.

To measure structural mobility, we computed the residue-wise root-mean-square fluctuation (RMSF) of backbone atoms. Structural changes over time, both for the *apo Sa*NsrF proteins and the ATP/Mg^2+^-bound form, were detected calculating the root-mean-square deviation of atomic positions (RMSD) compared to the initial structure. To describe the changes occurring at the level of the interface, we performed two analyses: (I) measurement of the distance between the center of mass of two residues located in opposite subunits at the center of the interface (S43 and S146); (II) H-bond analysis (i) in terms of the total number of interactions between two subunits (*Sa*NsrF_A_–*Sa*NsrF_B_) and between protein and ligands (*Sa*NsrF-(ATP-Mg^2+^)) and ii) residue-wise H-bound occupancy between residues of the two subunits (*Sa*NsrF_A_–*Sa*NsrF_B_), allowing to identify which residues perform more frequent H-bonds throughout the simulations. For this analysis, only H-bonds with the following criteria were considered: occupancy between specific donor and acceptor > 1%; H-bond present in at least two replicas of the same system; H-bonds between two residues with residue-wise occupancy > 10% in at least one system.

### Molecular docking of other NTPs

To predict the binding mode of other NTPs in complex with the *Sa*NsrF_WT_ dimer, molecular docking was performed. The starting points for these calculations were the five structures resulting from thermalization and equilibration steps, then used also for independent MD simulations replicas (production).

First, for each binding site a cubic grid of 20 Å length centered on the respective ATP molecule was built in the Maestro platform^92^, for a total of 10 different grids. Then, starting from the ATP structures, other NTPs were built (GTP, CTP and UTP) by modifying the nucleobase. The generated conformations were refined and scored with the Glide-Extra precision (XP) mode of Glide^93^. Only the best solution for each NTP in each grid was considered. The Coulomb interaction energy (ecoul) and the van der Waals energy (evdw), components of the XP GlideScore scoring function, were computed, and used to describe the enthalpic contribution of binding.

## Supplementary information

Supplementary Information.

## Data Availability

We upload the SAXS data and the corresponding model of *Sa*NsrF to the Small Angle Scattering Biological Data Bank (SASBDB)^54,55^, with the accession code SASDJR3.
