# Supplementary material for: Characterization of the nucleotide-binding domain NsrF from the BceAB-type ABC-transporter NsrFP from the human pathogen Streptococcus agalactiae
Source: Sci Rep. 2020 Sep 16;10:15208. doi: 10.1038/s41598-020-72237-7 (PMC7494861; doi:10.1038/s41598-020-72237-7)
Supplement: Supplementary file 1 — Supplementary Information. [file 41598_2020_72237_MOESM1_ESM.docx]

Supporting information

**Characterization of the Nucleotide-Binding Domain NsrF from the BceAB-type ABC-Transporter NsrFP from the Human Pathogen *Streptococcus agalactiae***

Fabia Furtmann^1*^, Nicola Porta^2*^, Dai Tri Hoang^1^, Jens Reiners^3^, Julia Schumacher^1^, Julia Gottstein^1^, Holger Gohlke^2,4^, Sander H. J. Smits^1,3#^

^1^Institute of Biochemistry, Heinrich-Heine-Universität Düsseldorf, Universitätsstraße 1, 40225 Düsseldorf, Germany

^2^Institute for Pharmaceutical and Medicinal Chemistry, Heinrich-Heine-Universität Düsseldorf, Universitätsstraße 1, 40225 Düsseldorf, Germany.

^3^Center for Structural Studies, Heinrich-Heine-Universität Düsseldorf, Universitätsstraße 1, 40225 Düsseldorf, Germany

^4^John von Neumann Institute for Computing (NIC), Jülich Supercomputing Centre (JSC), Institute of Biological Information Processing (IBI-7: Structural Biochemistry), Forschungszentrum Jülich GmbH, Wilhelm-Johnen-Straße, 52425 Jülich, Germany.

*These authors contributed equally

Keywords: antibiotic resistance, lantibiotics, nisin, ABC transporter, molecular dynamics simulations, structural model

Author ORCID
Nicola Porta: 0000-0002-6005-4372
Holger Gohlke: 0000-0001-8613-1447
Sander Smits: 0000-0003-0780-9251

^#^ Corresponding author: Sander Smits

Address: Universitätsstr. 1, 40225 Düsseldorf, Germany.

Phone: (+49) 211 81 12647; Fax: (+49) 211 81 15037

E-mail: sander.smits@hhu.de

**Materials and Methods**

***Multiangle Light Scattering (MALS)***

To determine the protein’s stoichiometry, Multiangle Light Scattering (MALS) was employed. Each protein was diluted in 100 mM HEPES at pH 8 and 300 mM NaCl. An Agilent 1260 HPLC system was used in combination with a triple-angle light scatter detector (miniDAWN TREOS II) and a differential refractive index detector (Optilab T-rEX) (both Wyatt Technology). *Sa*NsrF_WT_ at a concentration of 3 mg/mL were injected onto a Superdex 75 16/300 increase column with a flowrate of 0.6 mL/min. For analysis the ASTRA software package (Astra 7.1) (Wyatt Technology) was used (Fig. 1B).

***ATPase Activity Assay***

The ATPase activity of *Sa*NsrF_WT_ and *Sa*NsrF_H202A_ (diluted in 100 mM HEPES at pH 8, 100 mM NaCl) was examined by the Malachite Green Phosphate Assay at a protein concentration of 0.1 mg/mL that was initially undertaken at room temperature (20 °C). The initial conditions required a final Mg^2+^-concentration of 10 mM and an ATP concentration from 0 – 5 mM that was used for the reaction start.

Changes in protein’s activity were detected in a time range from 4 – 26 min by stopping a part of the reaction every two minutes, whereupon 18 min were chosen as an optimal time of incubation. An ATP (or NTP) concentration was set at 3 mM for following ATPase screenings. By the addition of EDTA (final concentration: 50 mM) a control was implemented in order to observe the process of autohydrolysis in a Mg^2+^-free solution.

To determine the maximal possible hydrolysis activity of *Sa*NsrF_WT_, the protein was exposed to various buffer systems including a citrate buffer for pH 4 and 5, MES for pH 6, HEPES for pH 7 and 8, TRIS for pH 7, 8, 9 and CAPS for pH 10 and 11 (100 mM for each). Moreover, the influence of the NaCl concentration on the ATPase-buffer was analysed by determining the ATP hydrolysis of the *Sa*NsrF_WT_ proteins in a buffer with 0, 100, 200, 300, 400, 500 and 1000 mM NaCl. Investigations were made concerning the cofactor choice of *Sa*NsrF_WT_ by introducing Ca^2+^, Mn^2+^, Zn^2+^, Fe^2+^ and Cu^2+^ instead of Mg^2+^ at a final concentration of 10 mM. An identical setup was performed without the addition of protein, which was used as a blank to encounter for autohydrolysis. The reaction including the optimized parameters was performed at 20 °C, 25 °C, 30 °C and 37 °C.

**Results**

***Activity of SaNsrF_WT_***

After successful purification, we functionally characterized *Sa*NsrF_WT_. To do so, we screened the following parameters on their influence on the ATP hydrolysis velocity: I) pH, II) salt concentration, III) nature of the divalent ion, and IV) temperature. This allowed us to obtain optimal conditions for our kinetic measurements

***pH dependency of SaNsrF_WT_***

We assayed the ATP hydrolysis of the *Sa*NsrF_WT_ protein at different pH conditions in order to determine the optimal buffer composition. Therefore, we used 100 mM of the following buffers: citrate at pH 4.0 – 5.0, MES at pH 6.0, HEPES at pH 7.0 – 8.0, TRIS at pH 7.0 – 9.0 and CAPS at pH 10.0 – 11.0.

We observed a large dependence on the pH of the buffer system (Figure 2A). ATP hydrolysis mediated by *Sa*NsrF_WT_ can only be observed in a pH range from 6.0 – 8.0, and the highest ATPase activity was reached in a HEPES buffer at pH 7.0. Interestingly, a reduction in activity of about 20 – 30 % is observed between HEPES (zwitterionic sulphonic acid) and TRIS (cationic primary amine) buffer systems although the pH was very similar (7.0 and 8.0, respectively).

***Influence of salt concentration on the activity of SaNsrF_WT_***

Next, we tested the influence of the ionic strengh on the activity of the *Sa*NsrF_WT_ protein. We used 100 mM HEPES at pH 7.0 as the optimal conditions for protein activity and varied the NaCl concentration ranging from 0 – 1 M in steps of 0.1 M (Figure 2B).

With increasing NaCl concentration, the hydrolytic activity strongly decreased. At a concentration of 1 M NaCl, 20% residual activity was recorded when compared to the maximum reached at 0 mM NaCl.

***Choice of Cofactor***

As a third optimization step, we examined the influence of the cofactor of *Sa*NsrF_WT_ on ATP hydrolysis. We replaced the 10 mM Mg^2+^ used so far with 10 mM Ca^2+^, Mn^2+^, Zn^2+^, Fe^2+^ or Cu^2+^, respectively. This revealed a clear dependency on the nature of the divalent ion where only for Mg^2+^ a reasonable hydrolytic activity was detected. Besides Mg^2+^, also Mn^2+^ was taken up as cofactor, with an ATPase activity of about a fourth of the maximally measured value. The other tested divalent ions did not significantly (e.g. Cu^2+^ caused about 15 % of the activity maximum) contribute to the ATPase activity of *Sa*NsrF_WT_ (Fig. S1C).

***Temperature dependence***

We assayed the ATPase activity of *Sa*NsrF_WT_ within a temperature range from 20 °C – 37 °C including the optimized parameters of 100 mM HEPES assay buffer at pH 7 with no added NaCl (see above). 10 mM Mg^2+^ were used to provide the protein with its cofactor. As illustrated in Figure 2D, the ATPase activity of *Sa*NsrF_WT_ was maximal at 30 °C. Further increase in the temperature resulted in a significant loss of activity as observed for 37 °C.

In summary, we varied several parameters of the ATPase activity assay in order to obtain the maximal hydrolytic activity for the *Sa*NsrF_WT_ protein. As a result, the optimized conditions were found to be 100 mM HEPES at pH 7 with 0 mM NaCl as an assay buffer. The reaction approach included the addition of 10 mM Mg^2+^ and was finally performed at a temperature of 30 °C. The reaction with the respective NTP was followed for an incubation time of 18 min, then stopped and measured. These optimized conditions were applied in all following experiments.

**
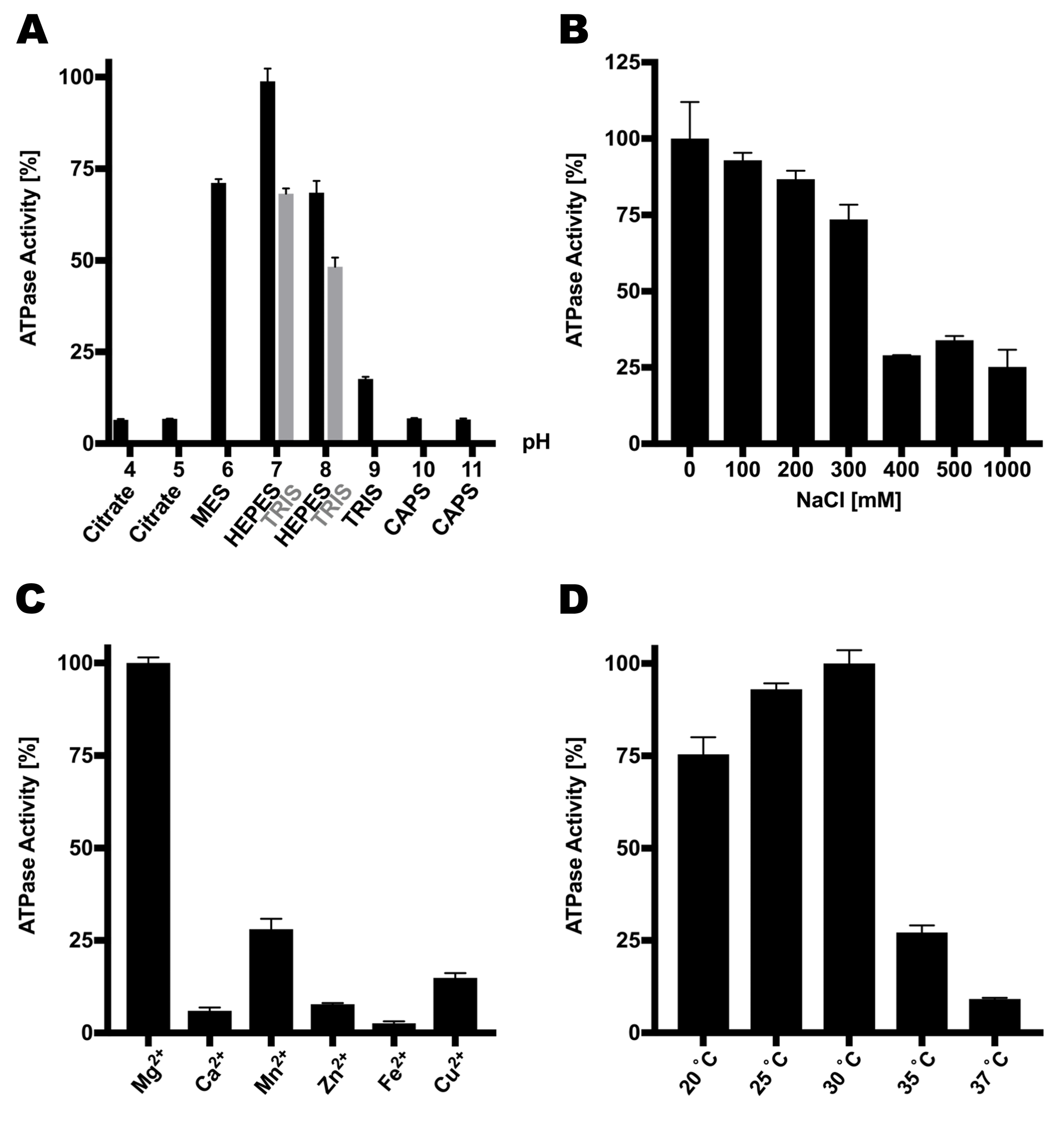
**

**Figure S1.** **Influence of ATPase activity of SaNsrF_WT_ by parameter variations.** **(A)** ATPase activity [%] of SaNsrF_WT_ dependent on pH and buffer system. 100 mM of citrate, MES, HEPES, TRIS and CAPS were diluted in ddH2O and adjusted to the respective pH. At pH 7 and 8 HEPES as well as TRIS were tested. **(B)** ATPase activity [%] of SaNsrF_WT_ dependent on concentrations of 0 mM to 1 M NaCl. SaNsrF_WT_ was diluted in 100 mM HEPES at pH 7 (0.1 mg/mL). **(C)** ATPase activity [%] of SaNsrF_WT_ dependent on twofold metal ions. SaNsrF_WT_ was exposed to 10 mM of Mg^2+^, Ca^2+^, Mn^2+^, Zn^2+^, Fe^2+^ or Cu^2+^. SaNsrF_WT_ was diluted in 100 mM HEPES at pH 7 (0.1 mg/mL). **(D)** ATPase activity [%] of SaNsrF_WT_ dependent on temperature (triple evaluation). A concentration of 3 mM ATP was applied on SaNsrF_WT_ (0.1 mg/mL; diluted in 100 mM HEPES at pH 7). The reaction was incubated at temperatures of 20 °C, 25 °C, 30 °C, 35°C and 37 °C and was stopped after 18 min and dyed as described in ATPase Activity Assay for 7 min. All experiments have been performed in at least three biological replicates and are represented as means ± s.d..

**
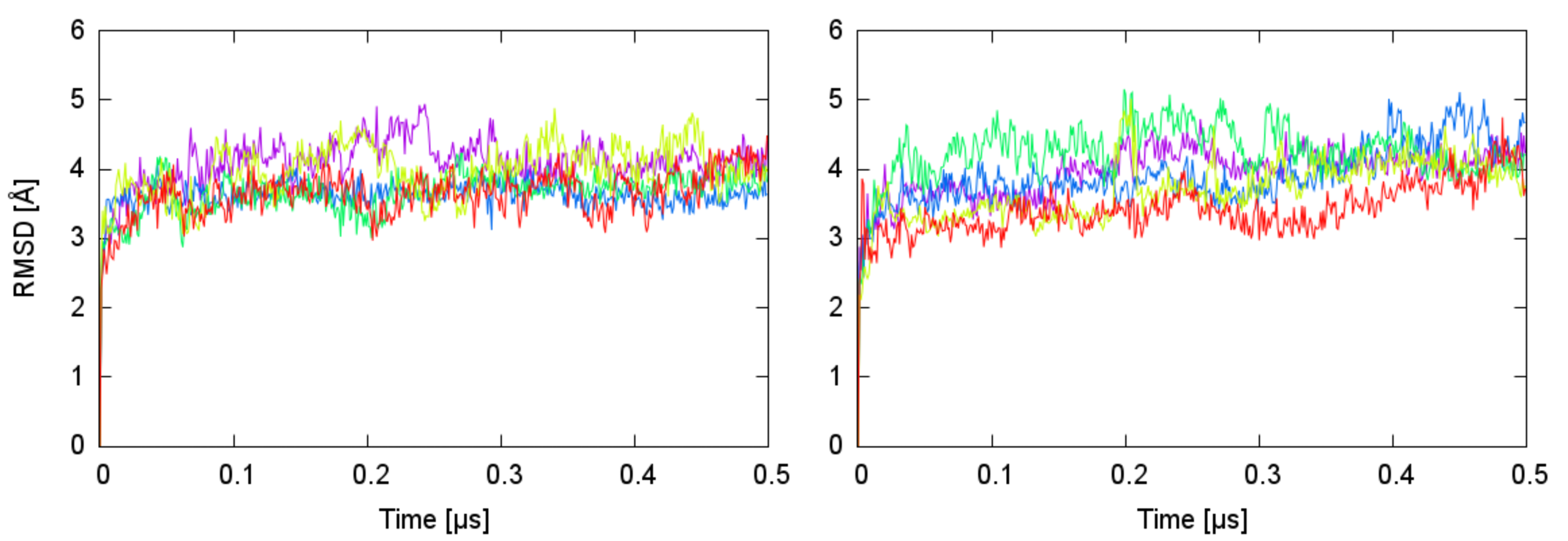
**

**Figure S2: Structural variability of *Sa*NsrF monomers.** The RMSD of backbone atoms was calculated after fitting the structures onto the 15% least mobile residues for each replica for *Sa*NsrF_WT_ (left) and for the *Sa*NsrF_H202A_ variant (right). The profiles are reported in a different color for each replica.


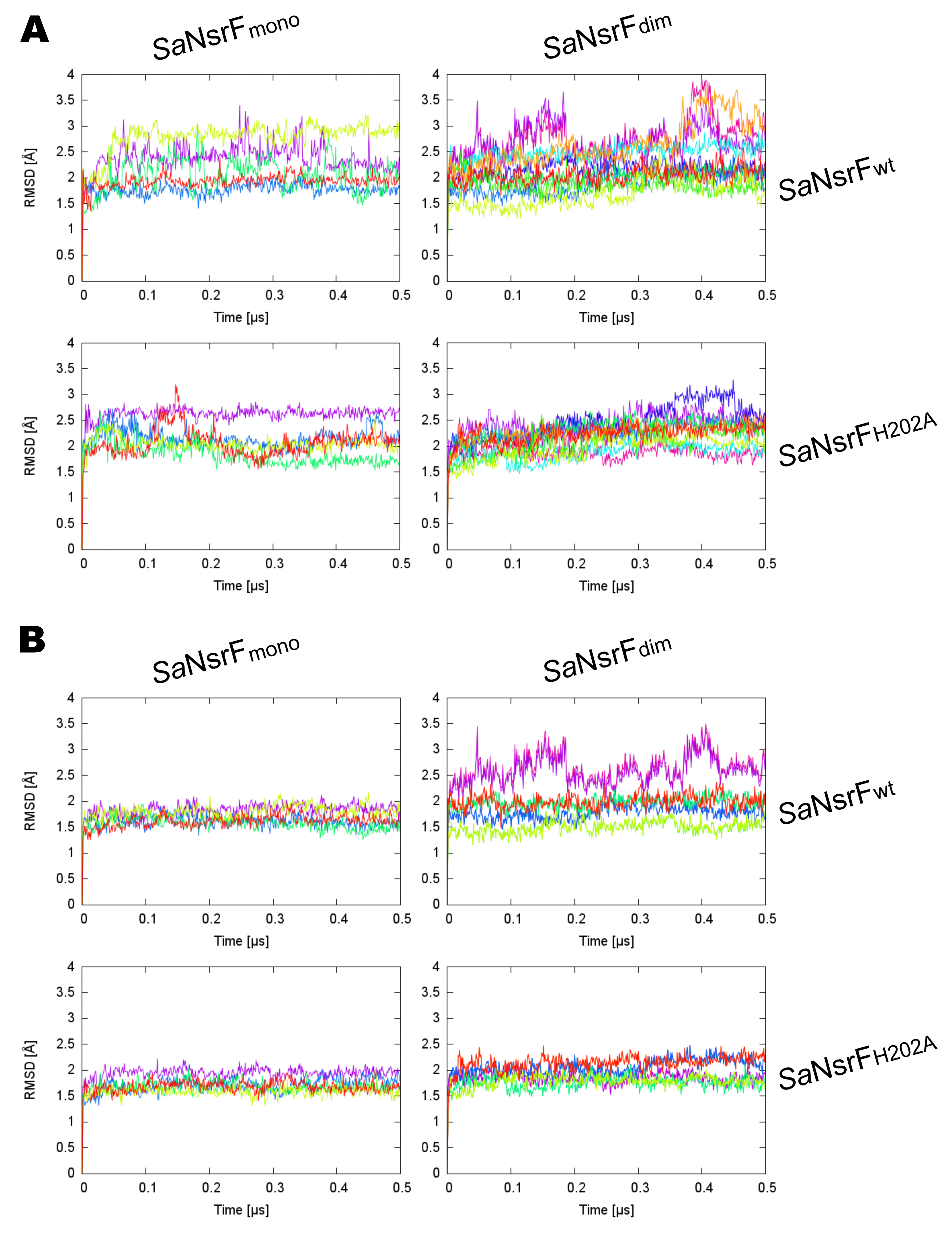


F**igure S3: Change of ATP molecule and Mg^2+^ ion positions with respect to the protein for different *Sa*NsrF systems. (A)** RMSD of ATP molecules; **(B)** RMSD of Mg^2+^ ions. The RMSD was calculated after fitting the structures onto the 15% least mobile protein residues for each replica. The profiles are shown in a different color for each molecule in each replica.


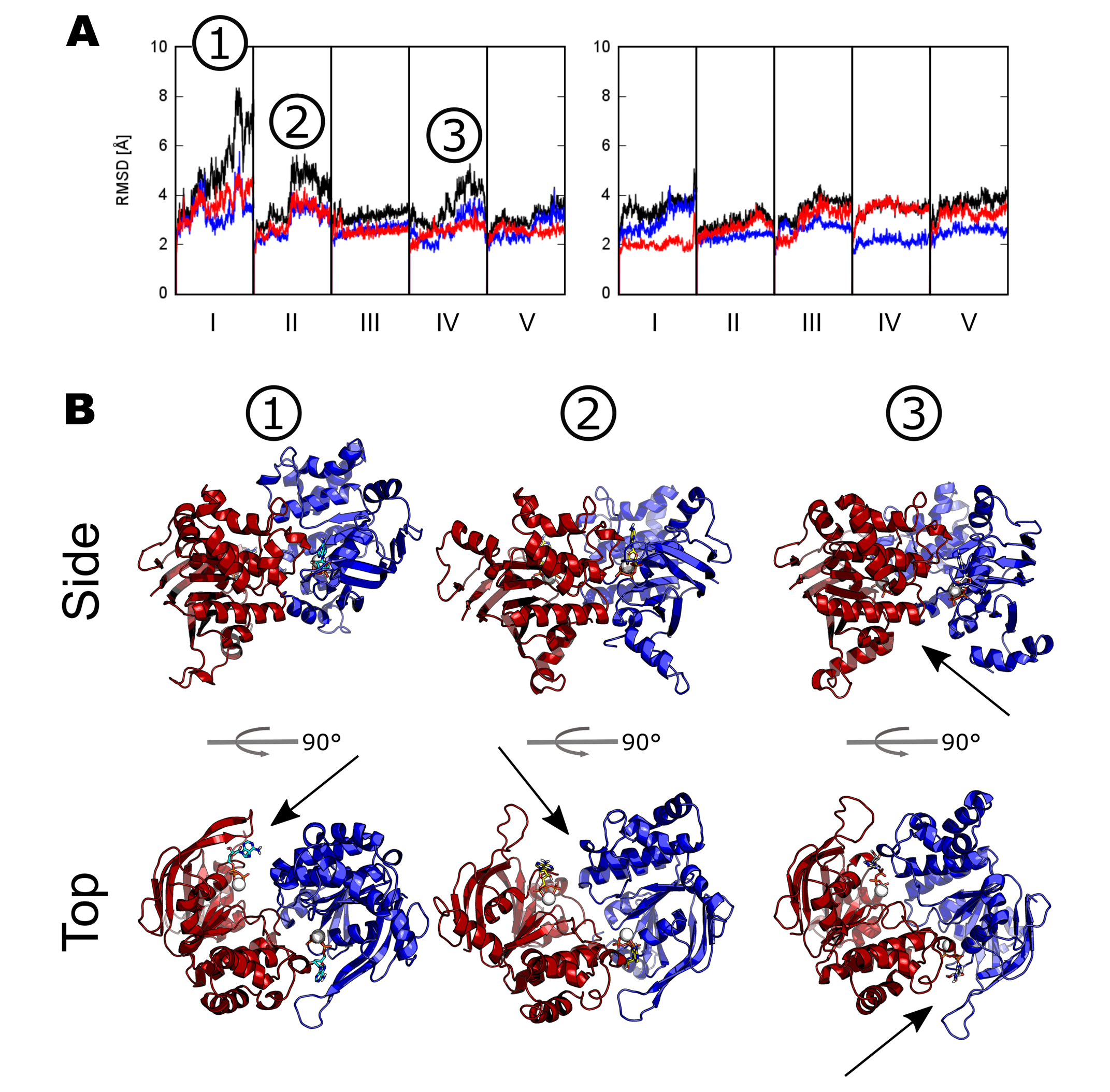


**Figure S4: Structural variability of *Sa*NsrF_WT_ and *Sa*NsrF_H202A_ dimers. (A)** The RMSD of backbone atoms was calculated for *Sa*NsrF_WT_ (left) and for the *Sa*NsrF_H202A_ variant (right) after fitting the structures onto the 15% least mobile residues for the whole dimer (black) and separately for either subunit A (red) or B (blue). Each box represents a replica of 0.5 µs simulation length (roman numbers). **(B)** Three representative structures of *Sa*NsrF_WT_ with higher RMSD values are displayed from two orientations. Arrows highlight the partial opening of the dimer interface.


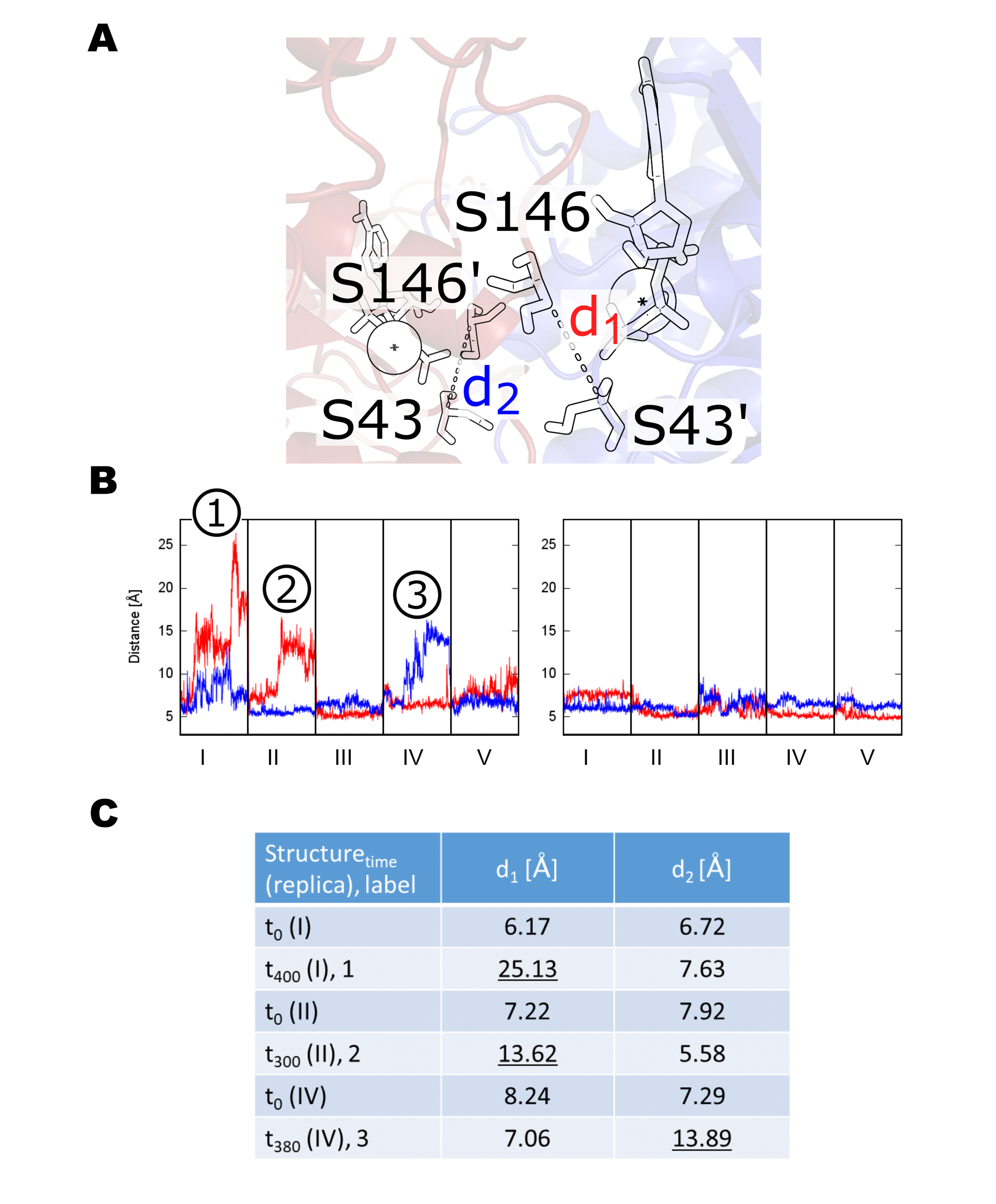


**Figure S5: Structural variability of dimers, expressed as distance between residues. (A)** Representation of the distances *d1* and *d2* between the residues S43 and S146 (centers of mass) located on different monomers. **(B)** Distances for *Sa*NsrF_WT_ dimer (left) and the *Sa*NsrF_H202A_ variant (right). Each box represents a replica of 0.5 µs simulation length (roman numbers). **(C)** Distances for three representative structures with higher RMSD values are reported; these structures are identical to the ones shown in Figure S4. The largest distance values, indicating the separation of the subunits, are underlined.

**
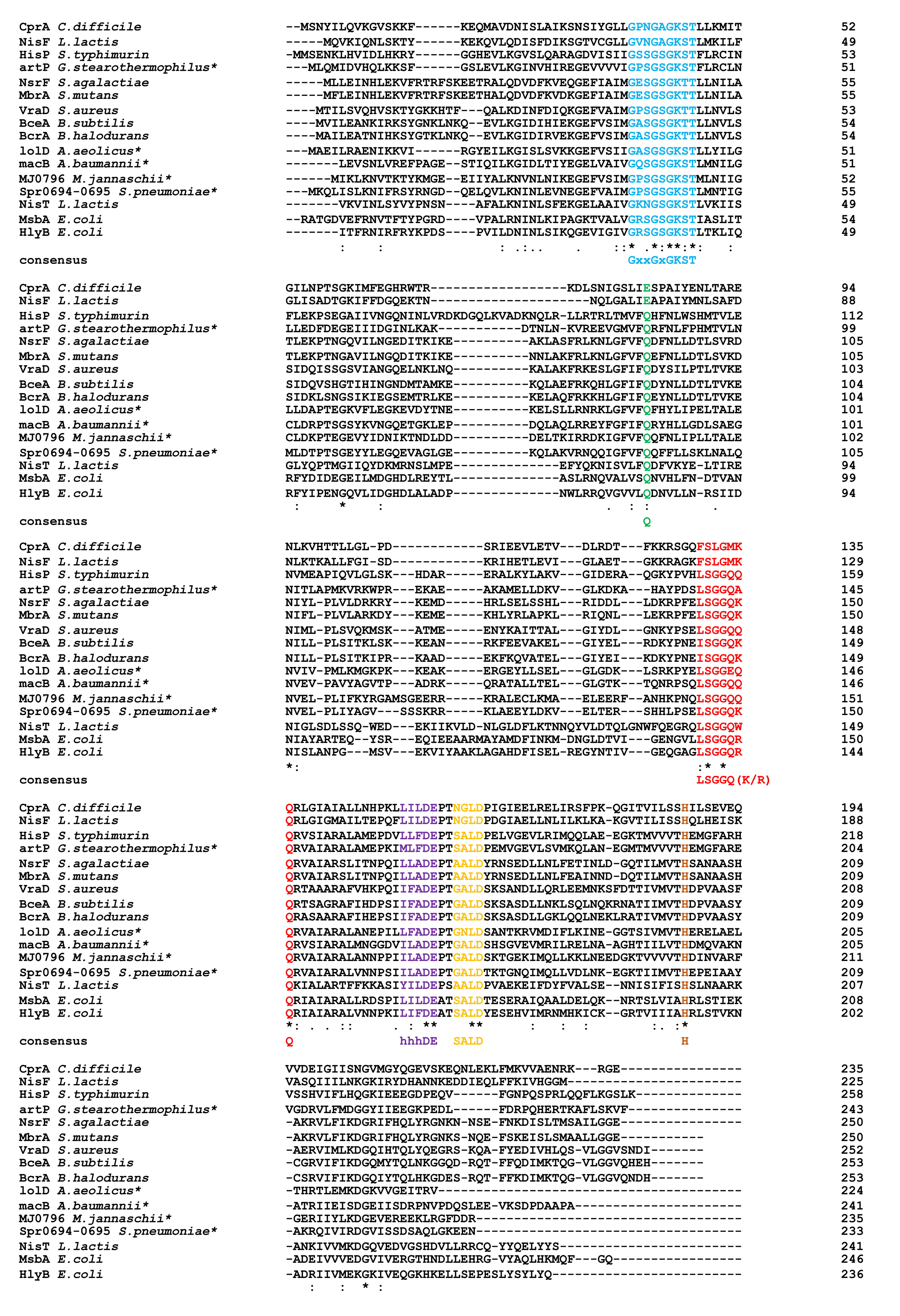
**

**Figure S6: Sequence alignment of different NBDs.** The sequence aligment was done with Clustal Omega ^1^. TopModel templates are marked with an asterix (*). The conserved motives are labeled in cyan (Walker A), green (Q-loop), red (C-loop), purple (Walker B), orange (D-loop) and brown (H-loop).

**Table S1: Five main templates used by the TopModel software for modeling of *Sa*NsrF.**

| **PDB ID:** | **Identity [%]** | **Similarity [%]** | **Coverage [%]** | **TM-Score [%]** | **Note** |
| --- | --- | --- | --- | --- | --- |
| 1F3O [a] | 37.7 | 88.4 | 88.0 | 91.9 | ADP/Mg^2+^-bound dimer, not functionally active assembly |
| 5XU1 [b] | 39.5 | 89.4 | 88.0 | 90.9 | Mg^2+^-bound dimer with TM domain |
| 2PCL [c] | 40.5 | 84.1 | 88.8 | 90.6 | Mg^2+^-bound dimer, not functionally active assembly |
| 5GKO [d] | 35.5 | 83.6 | 93.6 | 90.3 | Apo dimer with TM domain |
| 2OLI [e] | 30.9 | 76.1 | 93.2 | 89.2 | α-helical domain only |

[a] **^2^**; [b] **^3^**; [c] According to RCSB: “To be published”; [d] **^4^**; [e] According to RCSB: “To be published”.

# **Table S2: Consensus sequence of conserved motifs in the NBDs listed according to their occurrence from N- to C-terminus ^5^**. For a graphical representation see Fig. 3B.

| **Motif** | **Consensus sequence** | ***Sa*NsrF** |
| --- | --- | --- |
| A-loop | (F/K)xY | 10-KVF-12 |
| Walker A or P-loop | GxxGxGK(S/T) | 41-GESGSGKT-48 |
| Q-loop | hV(S/P)Q | 90-FVFQ-93 |
| X-loop | TRVGDKGTQ | 137-LLDKRP-142 |
| Signature motif or C-loop | LSGGQ(K/R)Q | 145-LSGGQKQ-151 |
| Walker B | hhhhDE | 165-ILLADE-170 |
| D-loop | SALD | 173-AALD-176 |
| H-loop | hAHRL | 200-VTH^202^SA-204 |

**Table S3: Overall SAXS Data**

| **SAXS Device** | Xenocs Xeuss 2.0 with Q-Xoom |
| --- | --- |
| **Data collection parameters** |  |
| Detector | PILATUS 3 R 300K windowless |
| Detector distance (m) | 0.550 |
| Beam size (mm x mm) | 0.8 x 0.8 |
| Wavelength (Å) | 1.54 (Cu Source) |
| Sample environment | Low Noise Flow Cell, 1 mm ø |
| *s* range (nm^-1^)^‡^ | 0.18 – 6.0 |
| Temperature (K) | 288 |
| Exposure time per frame (min) | 10 |
| **Sample** | ***Sa*NsrF_WT_** |
| Mode of measurement | static |
| Protein concentration (mg/ml) | 0.5 – 4.2 |
| **Structural parameters** |  |
| *I(0)* from P(r) | 0.023 |
| *R*_g_ (real-space from P(r)) (nm) | 2.46 |
| *I*(0) from Guinier fit | 0.023 |
| *s-range* for Guinier fit (nm^-1^) | 0.23 – 0.54 |
| *R*_g_ (from Guinier fit) (nm) | 2.40 |
| *D*_max_ (nm) | 7.90 |
| POROD volume estimate (nm^3^) | 64.37 |
| **Molecular mass (kDa)** |  |
| From I(0) | 31.85 |
| From MoW2 ^6^ | 33.36 |
| From Vc ^7^ | 38.51 |
| From POROD | 40.23 |
| From sequence | 30.86 |
| **Structure Evaluation** |  |
| Ambimeter score | 0.6990 |
| Crysol χ^2^ | 1.16 |
| **Software** |  |
| ATSAS Software Version ^8^ | 3.0.1 |
| Primary data reduction | PRIMUS ^9^ |
| Data processing | GNOM ^10^ |
| *Ab initio* modelling | GASBOR ^11^ |
| Superimposing | SUPCOMB ^12^ |
| Structure evaluation | AMBIMETER ^13^ / CRYSOL ^14^ |
| Model visualization | PyMOL ^15^ |

‡s = 4π sin(θ)/λ, 2θ – scattering angle

**
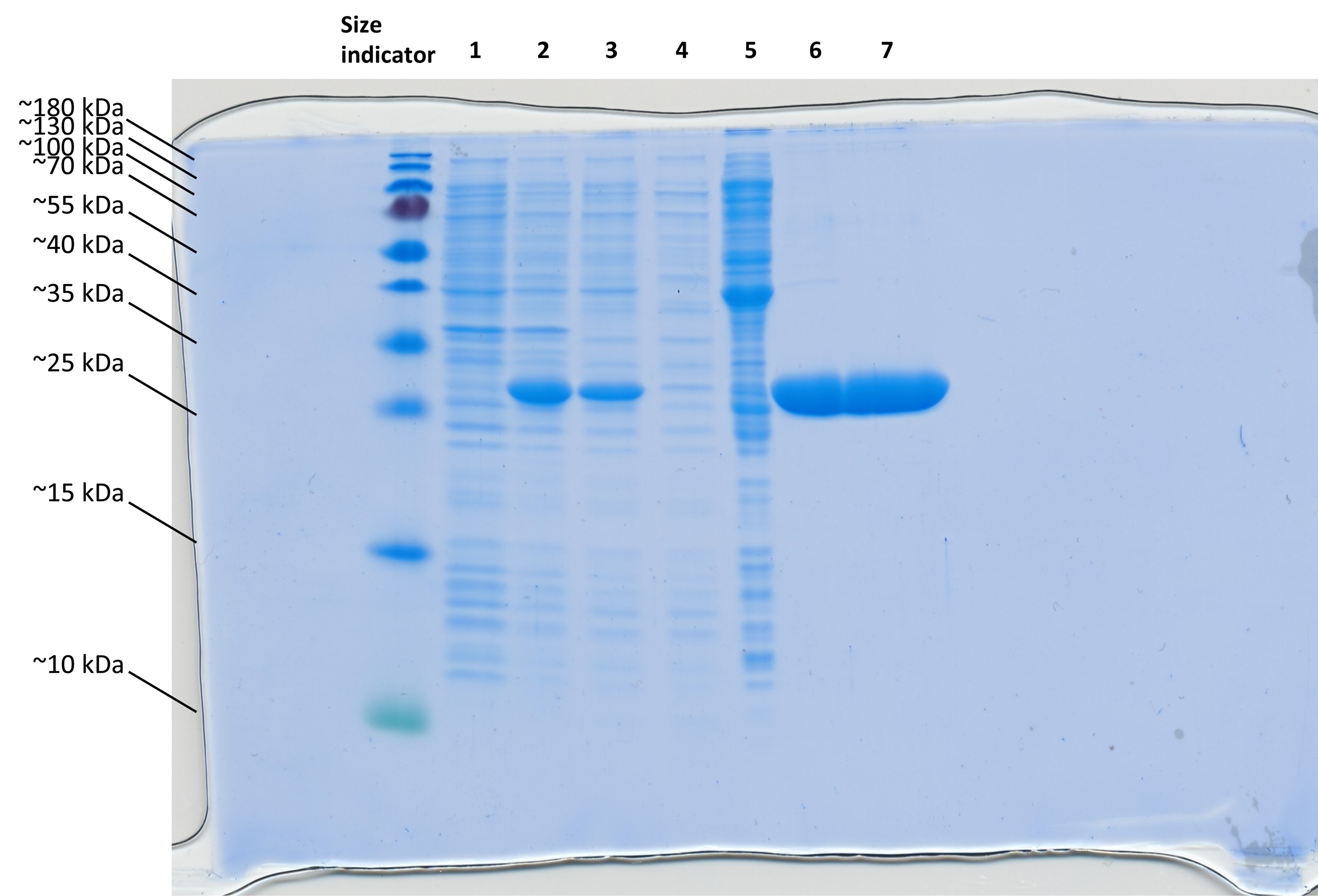
**

**Original Figure 1.** **Purification and SEC-MALS of *Sa*NsrF_WT_.** **(A)** SDS-PAGE of the *Sa*NsrF_WT_ purification progress. PageRuler Prestained Protein Ladder (size indicator; 10 to 180 kDa), *E. coli* strain before IPTG induction (1), *E. coli* strain after IPTG induction (2), IMAC load (3), IMAC flow-through (4), IMAC wash-fraction (5), IMAC eluate (6), SEC eluate (7).

**References:**

1 Madeira, F. *et al.* The EMBL-EBI search and sequence analysis tools APIs in 2019. *Nucleic Acids Res* **47**, W636-W641, doi:10.1093/nar/gkz268 (2019).

2 Yuan, Y. R. *et al.* The crystal structure of the MJ0796 ATP-binding cassette. Implications for the structural consequences of ATP hydrolysis in the active site of an ABC transporter. *J Biol Chem* **276**, 32313-32321, doi:10.1074/jbc.M100758200 (2001).

3 Yang, H. B. *et al.* Structure of a MacAB-like efflux pump from *Streptococcus pneumoniae*. *Nat Commun* **9**, 196, doi:10.1038/s41467-017-02741-4 (2018).

4 Okada, U. *et al.* Crystal structure of tripartite-type ABC transporter MacB from *Acinetobacter baumannii*. *Nat Commun* **8**, 1336, doi:10.1038/s41467-017-01399-2 (2017).

5 Szollosi, D., Rose-Sperling, D., Hellmich, U. A. & Stockner, T. Comparison of mechanistic transport cycle models of ABC exporters. *Biochim Biophys Acta Biomembr* **1860**, 818-832, doi:10.1016/j.bbamem.2017.10.028 (2018).

6 Fischer, H., Neto, M. D., Napolitano, H. B., Polikarpov, I. & Craievich, A. F. Determination of the molecular weight of proteins in solution from a single small-angle X-ray scattering measurement on a relative scale. *J Appl Crystallogr* **43**, 101-109, doi:10.1107/S0021889809043076 (2010).

7 Rambo, R. P. & Tainer, J. A. Accurate assessment of mass, models and resolution by small-angle scattering. *Nature* **496**, 477-481, doi:10.1038/nature12070 (2013).

8 Franke, D. *et al.* ATSAS 2.8: a comprehensive data analysis suite for small-angle scattering from macromolecular solutions. *J Appl Crystallogr* **50**, 1212-1225, doi:10.1107/S1600576717007786 (2017).

9 Konarev, P. V., Volkov, V. V., Sokolova, A. V., Koch, M. H. J. & Svergun, D. I. PRIMUS: a Windows PC-based system for small-angle scattering data analysis. *J Appl Crystallogr* **36**, 1277-1282, doi:10.1107/S0021889803012779 (2003).

10 Svergun, D. I. Determination of the Regularization Parameter in Indirect-Transform Methods Using Perceptual Criteria. *J Appl Crystallogr* **25**, 495-503, doi:Doi 10.1107/S0021889892001663 (1992).

11 Svergun, D. I., Petoukhov, M. V. & Koch, M. H. Determination of domain structure of proteins from X-ray solution scattering. *Biophys J* **80**, 2946-2953, doi:10.1016/S0006-3495(01)76260-1 (2001).

12 Kozin, M. B. & Svergun, D. I. Automated matching of high- and low-resolution structural models. *J Appl Crystallogr* **34**, 33-41, doi:Doi 10.1107/S0021889800014126 (2001).

13 Petoukhov, M. V. & Svergun, D. I. Ambiguity assessment of small-angle scattering curves from monodisperse systems. *Acta Crystallogr D Biol Crystallogr* **71**, 1051-1058, doi:10.1107/S1399004715002576 (2015).

14 Svergun, D., Barberato, C. & Koch, M. H. J. CRYSOL - A program to evaluate x-ray solution scattering of biological macromolecules from atomic coordinates. *J Appl Crystallogr* **28**, 768-773, doi:Doi 10.1107/S0021889895007047 (1995).

15 The PyMOL Molecular Graphics System, Version 2.0 Schrödinger, LLC.
